# Simultaneous deletion of ORMDL1 and ORMDL3 proteins disrupts immune cell homeostasis

**DOI:** 10.3389/fimmu.2024.1376629

**Published:** 2024-04-23

**Authors:** Livia Demkova, Viktor Bugajev, Miroslava K. Adamcova, Ladislav Kuchar, Srdjan Grusanovic, Meritxell Alberich-Jorda, Petr Draber, Ivana Halova

**Affiliations:** ^1^ Laboratory of Signal Transduction, Institute of Molecular Genetics of the Czech Academy of Sciences, Prague, Czechia; ^2^ Laboratory of Hemato-Oncology, Institute of Molecular Genetics of the Czech Academy of Sciences, Prague, Czechia; ^3^ Research Unit for Rare Diseases, Department of Pediatrics and Inherited Metabolic Disorders, First Faculty of Medicine, Charles University and General University Hospital in Prague, Prague, Czechia

**Keywords:** lymphocyte, B cells, sphingolipids, ORMDL proteins, spleen

## Abstract

ORMDL3 is a prominent member of a family of highly conserved endoplasmic reticulum resident proteins, ORMs (ORM1 and ORM2) in yeast, dORMDL in *Drosophila* and ORMDLs (ORMDL1, ORMDL2, and ORMDL3) in mammals. ORMDL3 mediates feedback inhibition of *de novo* sphingolipid synthesis. Expression levels of ORMDL3 are associated with the development of inflammatory and autoimmune diseases including asthma, systemic lupus erythematosus, type 1 diabetes mellitus and others. It has been shown that simultaneous deletions of other ORMDL family members could potentiate ORMDL3-induced phenotypes. To understand the complex function of ORMDL proteins in immunity *in vivo*, we analyzed mice with single or double deletions of *Ormdl* genes. In contrast to other single and double knockouts, simultaneous deletion of ORMDL1 and ORMDL3 proteins disrupted blood homeostasis and reduced immune cell content in peripheral blood and spleens of mice. The reduced number of splenocytes was not caused by aberrant immune cell homing. A competitive bone marrow transplantation assay showed that the development of *Ormdl1^-/-^*/*Ormdl3^-/-^* B cells was dependent on lymphocyte intrinsic factors. Highly increased sphingolipid production was observed in the spleens and bone marrow of *Ormdl1^-/-^*/*Ormdl3^-/-^* mice. Slight, yet significant, increase in some sphingolipid species was also observed in the spleens of *Ormdl3^-/-^* mice and in the bone marrow of both, *Ormdl1^-/-^* and *Ormdl3^-/-^* single knockout mice. Taken together, our results demonstrate that the physiological expression of ORMDL proteins is critical for the proper development and circulation of lymphocytes. We also show cell-type specific roles of individual ORMDL family members in the production of different sphingolipid species.

## 1 Introduction

Autoimmune diseases, such as type 1 diabetes mellitus (T1D), rheumatoid arthritis (RA), psoriasis, multiple sclerosis (MS), inflammatory bowel diseases (IBD) and systemic lupus erythematosus (SLE), are diseases in which the body is attacked by its own immune system. The exact mechanism of their development is unknown but a combination of genetic and environmental factors has been implicated. The incidence of autoimmune diseases has risen sharply in recent decades (1, 2). Elucidating the mechanism of their development and identifying potential therapeutic targets are therefore, important goals in present-day research.

ORMDL3 is a prominent member of a family of highly conserved transmembrane endoplasmic reticulum (ER) proteins, ORMs (ORM1 and ORM2) in yeast, dORMDL in *Drosophila* and ORMDLs (ORMDL1, ORMDL2, and ORMDL3) in mammals (3). It has been identified as a potent inhibitor of the serine palmitoyltransferase (SPT) enzyme complex, which is responsible for the condensation of acyl-coenzyme A with serine, the first and rate-limiting step in *de novo* sphingolipid synthesis (3–5). The importance of ORMDL3 in the control of SPT enzymatic activity was confirmed just recently when the structure of the SPT enzymatic complex with ORMDL3 was solved (6, 7). Production of sphingolipids is controlled by a feedback loop, when the ORMDL-SPT inhibitory complex is stabilized by direct binding of ceramides (8, 9). Another feedback loop acts through sensing of increased levels of sphingosine-1-phosphate (S1P) by the S1P receptor 1 (S1PR1). Activation of the S1P-S1PR1 signaling axis is able to stabilize ORMDL expression (10). From ORMDL family members, ORMDL3 deletion appears to cause the highest elevation in sphingolipid levels. This elevation is further potentiated by the concurrent absence of other family members, ORMDL1 (11, 12) or ORMDL2 (13), depending on the cell type. Lone absence of ORMDL1 or ORMDL2 has no effect on sphingolipid levels in studied cells (11–13). However, it cannot be ruled out that the effect would be different in other tissues or cell types.

Proper regulation of sphingolipid levels is crucial for maintaining tissue homeostasis and its dysregulation leads to several diseases in humans (14, 15). Inability to synthesize sphingolipids leads to the lethal phenotype seen in mice with SPT subunits knockouts (KOs) (16–18). An uncontrolled increase of SPT activity is probably responsible for the inability to generate viable mice with all ORMDL proteins deleted simultaneously (11, 12). Currently, there is only one study, which describes a viable cell line with a simultaneous deletion of all three *ORMDL* genes (19). In addition to their well-established role in sphingolipid biosynthesis, ORMDL proteins were also identified as important regulators of ER-stress responses and autophagy (20–22). However, the direct mechanism of how ORMDL proteins regulate these processes has still not been fully elucidated.

Several studies linked single nucleotide polymorphisms (SNPs) in the locus of 17q12 – q21 chromosomal region with the development of inflammatory and autoimmune diseases (23–27). *ORMDL3* is one of the genes that resides in this locus. The most studied disease connected with SNPs in the abovementioned locus is early-onset childhood asthma, in which increased expression of ORMDL3 has been identified (25, 26). An association between elevated ORMDL3 protein levels and asthma has not only been observed in humans but also in experimental mice (21, 28, 29) and even in *Drosophila* (30). However, the exact involvement of ORMDL3 in the development of asthma is still not completely understood. The more studies that are published, the more controversial the topic becomes (reviewed in (31)).

Genetic variation studies identify SNPs as risk factors of specific complex diseases, yet they do not provide a direct causal link between the pathogenesis of a disease. The SNP that most strongly associated with childhood asthma and was linked with increased transcript levels of the *ORMDL3* gene was rs7216389 (25). The SNP lies within a chromosomal locus that not only encompasses *ORMDL3*, but also other genes, such as gasdermin B (*GSDMB*), zona pellucida binding protein 2 (*ZPBP2*) and IKAROS family zinc finger 3 (*IKZF3*), suggesting that variations in *ORMDL3* expression may not be the sole determinants of disease susceptibility (25, 26, 32). SNPs within the same genetic region have also been associated with the risk of SLE, T1D and IBD. (20, 23, 24, 27). The impact that SNPs in non-coding chromosomal regions exert on transcript levels of genes is dependent on the steady state expression of a gene and its epigenetic regulation (25, 26). Both of these factors are tissue and cell-type specific (5, 25, 26), contributing to the differing effects varying gene expression has on individual cells and disease states.

Increased *ORMDL3* gene expression was detected early in life in cord blood mononuclear cells sampled from donors with asthma-risk SNPs within the 17q12 – q21 locus, suggesting that ORMDL3 may play a role in early immune maturation (33). Transcript levels of both, *ORMDL3* and *GSDMB*, were increased in primary immune cell types isolated from the peripheral blood of donors carrying the rs7216389 SNP. The highest *ORMDL3* mRNA levels were noted in naïve CD4^+^ T cells and B cells, while no changes were noted in monocytes and dendritic cells (34). ORMDL3 is highly expressed in murine airway epithelial cells and murine models of asthma showed that its expression is further induced following an allergen challenge (21, 22). In Fas^lpr/lpr^ lupus murine model, the spleens of the mice exhibited significantly increased total ORMDL proteins. Correspondingly, patients with SLE displayed increased *ORMDL3* transcript levels in peripheral blood mononuclear cells (20). Although clinical association studies correlated an increased expression of *ORMDL3* with risk of Crohn’s disease (23), gut biopsy samples from Crohn’s disease patients showed comparable levels of *ORMDL3* expression with controls (35). Decreased levels of *ORMDL3* mRNA were detected in leukocytes isolated from the peripheral blood of children with T1D compared to healthy children (36). *ORMDL3* was also identified as an obesity-related gene, and its expression was negatively correlated with body mass index (37). Consistent with these findings, *ORMDL3* expression was significantly downregulated in pancreatic islet cells isolated from overweight/obese human donors compared with lean controls (38).

We identified ORMDL3 as a negative regulator of proinflammatory cytokine and prostaglandin production in IgE-antigen stimulated mast cells (13, 39). This phenotype was further potentiated by the subsequent absence of ORMDL1 (12) or ORMDL2 (13). Recently, we showed that ORMDL3 influences not only sphingolipid but also leukotriene synthesis through a direct interaction with 5-lipoxygenase, an enzyme that mediates the conversion of arachidonic acid to leukotriene signaling mediators (40). With several exceptions (11, 20), the function of ORMDL proteins in health and disease has mostly been studied in relation to asthma [reviewed in (31)]. Therefore, research has focused on the study of increased ORMDL3 levels. To elucidate the role of decreased levels of ORMDL proteins in mammals and their potential role in the development of autoimmune diseases, we produced mice lacking individual ORMDL proteins, as well as all the possible double knockouts (dKOs) using CRISPR/Cas9 genome editing technology and subsequent cross-breeding. We showed that *Ormdl1^-/-^*/*Ormdl3^-/-^* (O1/3dKO) mice have smaller spleens with reduced immune cell content and altered blood homeostasis. Sphingolipid production was increased in the spleen and bone marrow (BM) of *Ormdl3^-/-^* (O3KO) and O1/3dKO mice and in BM of *Ormdl1^-/-^* (O1KO) mice. We proved that physiological levels of ORMDL proteins are important for immune cell development.

## 2 Materials and methods

### 2.1 Mice

The generation of single and double KO mice was described preciously (12, 13). All work with animals was conducted in accordance with the Institute of Molecular Genetics guidelines (permit number 12135/2010-17210) and national guidelines (2048/2004-1020). All experiments performed using mice were approved by the ethical committee of the Institute of Molecular Genetics and were conducted in accordance with the ARRIVE guidelines. Mice were housed in top-filter cages and fed a standard diet with freely available water and food at pathogen-free animal facilities at the Institute of Molecular Genetics. Age- and sex-matched mice were used for the purposes of this study. The experiments, where the results were affected by gender (mice weight, spleen size, red blood cell (RBC) numbers and proportions), are presented in gender separated figures. In all other cases, the results obtained using male and female mice were pooled after we confirmed they are independent of gender. The ratio of male and female was maintained in all experiments.

### 2.2 Antibodies, reagents and equipment

All antibodies, chemicals, mouse strains, commercial kits, software and important laboratory instruments are listed in **Supplementary Tables 1**, **2**.

### 2.3 Cell preparation and flow cytometry analysis

Mice of different genotypes were sacrificed, immune cells were isolated from the spleen, blood and BM of femurs and tibias. Erythrocytes were lysed in ammonium-chloride-potassium (ACK) lysis buffer. Cell suspensions were filtered through 70 μM strainers to remove cellular debris and washed in phosphate-buffered saline (PBS). Cells were stained on ice for 30 minutes with fluorescent-conjugated antibodies diluted in appropriately titrated concentrations. All antibodies used are listed in **Supplementary Table 1**. Labeled cells were analyzed on Symphony flow cytometer (BD Bioscience). Data were obtained using Diva software (BD Bioscience) and analyzed using FlowJo Software.

### 2.4 Histology

Spleens were isolated from 6 week-old mice, washed in PBS, fixed overnight in 4% formaldehyde solution and stored in 70% ethanol prior to processing. The specimen were processed for 12 hours in a HistoCore PEGASUS Plus (Leica Biosystems) tissue processor using ethanol and xylene for dehydration and histowax paraffin for impregnation. Following embedding in paraffin, the specimen were sectioned into 5 µm samples and transferred onto microscope slides. The samples were deparaffinized and rehydrated in xylene and serial dilutions of ethanol prior to staining with hematoxylin for 5 minutes and counterstaining with eosin for 3 minutes. The stained specimen were visualized on a Leica DM6000 widefield microscope using a 20x/0.70 objective for tile scan images and 10x/0.40 objective for single images.

### 2.5 Competitive transplantation assay

BM donor cells (5x10^6^) isolated from WT or O1/3dKO mice were transplanted into lethally irradiated CD45.1 congenic mice (recipient) together with support WT CD45.1 congenic BM cells (support, 5x10^6^). To assess short term hematopoietic reconstitution, recipient blood was analyzed 6 weeks after transplantation for the presence of newly developed WBC by flow cytometry. Long term repopulation abilities were analyzed 12 weeks post transplantation. Recipient mice were sacrificed and the presence of newly developed immune cells was analyzed in blood, BM and spleen by flow cytometry.

### 2.6 Homing of immune cells

Single cell suspensions of WT and O1/3dKO splenocytes were prepared as described above. Cells were labeled with CellTrace™ CFSE or Far Red, respectively (Invitrogen) according to the manufacturer´s instructions. Cells were washed and mixed in 1:1 ratio (5x10^6^ cells of each cell type) in 150 μl of sterile PBS. Cell suspensions were injected into tail veins of recipient mice (WT or O1/3dKO). Mice were sacrificed 24 hours later and cells were isolated from the spleens and BM. Single cell suspensions were labeled with fluorescently conjugated anti-B220 antibody to distinguish B cells and analyzed by flow cytometry for the presence of CellTrace-positive cells. The absolute number of homed cells in each organ was calculated based on frequencies of CellTrace-positive cells and organ cell counts.

### 2.7 Blood analysis

Blood was collected by submandibular bleeding into EDTA-coated tubes and analyzed using BC5300 Vet auto Hematology Analyzer (Mindray Bio-Medical Electronics Co., Ltd.).

### 2.8 Sphingolipid measurements

The extraction of sphingolipids and their analysis by tandem mass spectrometry (LC-ESI-MS/MS) was performed as described previously (13). Briefly, splenocytes or BM cells (6 × 10^6^) were sonicated. Protein concentrations were measured using a Pierce BCA protein assay kit according to the manufacturer’s instructions. Aliquots of 40 µg of proteins were transferred into glass vials containing internal standards (d17:1 sphingosine [Cat. No. 860640P] and d18:1/17:0 ceramide [Cat. No. 860517P], Avanti Polar Lipids) and mixed with 1 ml of 2:1 (v/v) chloroform/methanol solution. The samples were incubated for 60 minutes at room temperature under gentle agitation and filtered through Millex LH 0.45 µm filters (Merck Millipore) prior to LC-ESI-MS/MS analysis.

### 2.9 Statistical analysis

All statistical analysis was performed using GraphPad Prism software version 7.3. Data are presented as mean ± standard error of the mean (SEM) of at least three independent experiments. Data were tested for normality using the Shapiro-Wilk normality test. Comparison between two groups was analyzed by unpaired two-tailed Student’s *t*-test. Comparison of more than two groups was evaluated using one-way analysis of variance (ANOVA) with Tukey’s posttest for normally distributed data or Kruskal-Wallis test with Dunn’s posttest for non-parametric data as indicated in the figure legend. *P*-values of < 0.05 were considered to be statistically significant (*P < 0.05; **P < 0.01; ***P < 0.001).

## 3 Results

### 3.1 Mice with simultaneous deletion of ORMDL1 and ORMDL3 proteins present reduced numbers of immune cells in the spleen

**Figure 1:**
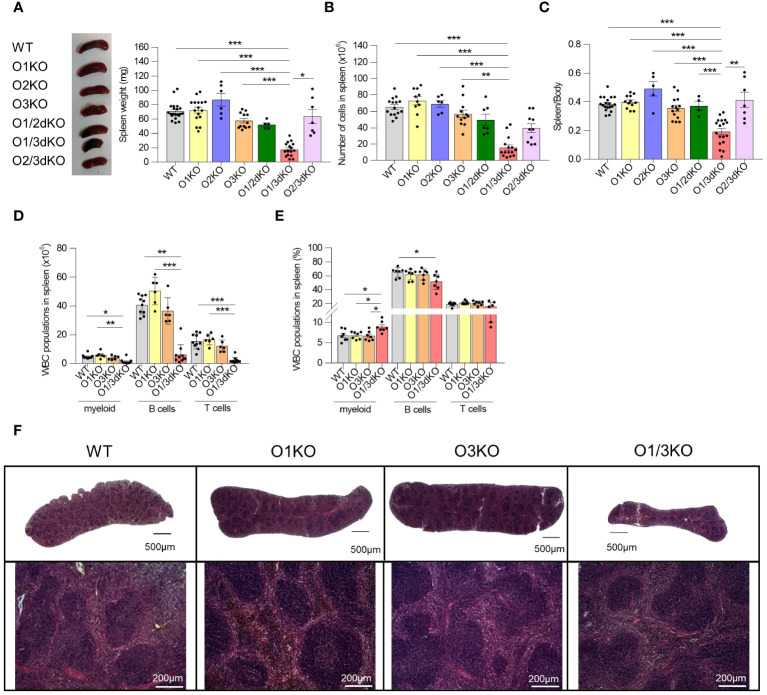
Simultaneous loss of ORMDL1 and ORMDL3 proteins reduces the number of immune cells in the spleen. **(A)** Representative image and statistical evaluation of spleen weight of 6 week-old female mice of different genotypes, (n ≥ 4). **(B)** Number of cells in spleen (n ≥ 6). **(C)** Ratio of spleen/body weight. Amount **(D)** and proportion **(E)** of different white blood cells (WBC) isolated from spleens (n ≥ 5). **(F)** Hematoxylin and eosin stained spleens isolated from 6 week-old mice. The upper panel shows stitched images of full-length histological sections of murine spleens, while the lower panel shows an enlarged portion of the respective spleen. Data were analyzed by one-way ANOVA with Tukey´s post-test. (*P < 0.05, **P < 0.01 and *** P < 0.001). Each dot represents individual biological replicates. WT, *Ormdl1^-/-^* (O1KO), *Ormdl3^-/-^* (O3KO), *Ormdl1^-/-^/Ormdl3^-/-^* (O1/3dKO).

Suppressed expression of ORMDL3 has been associated with the increased occurrence of autoimmune diseases (23, 24, 26). Deletion of the ORMDL3 protein in experimental animals led to a slight reduction of B cell numbers in their spleens (20). It has been shown that changes in phenotype observed in O3KO mice or cells can be potentiated by the simultaneous deletion of ORMDL1 or ORMDL2 proteins (11–13). To find out if this also applies to B cell development and homeostasis, we took advantage of our recently prepared single and double *Ormdl* KO mice (12, 13). In contrast to single KO and other double KO mice, O1/3dKO mice are smaller in size (11–13). We observed that spleen sizes of O1/3dKO mice were markedly reduced with a diminished immune cell content (**Figures 1A, B**). This was not a consequence of lower murine body weights of the mice as was evident from reduced spleen/body weight ratios (**Figure 1C**). Unlike in female mice, significantly smaller spleens were also observed in O3KO and O2/3dKO male mice (**Supplementary Figure 1A**). However, in these mice, the number of immune cells was not altered (**Supplementary Figure 1B**). The reduced spleen weight observed in O3KO and O2/3dKO males correlated with a lower body weight of the mice as was indicated by the unchanged spleen/body weight ratios (**Supplementary Figure 1C**).

Since the observed decrease of immune cell numbers was most pronounced in O1/3dKO mice, we decided to focus our study on the role of ORMDL proteins in B cell development and homeostasis using O1/3dKO mice, their single KO littermates (O1KO and O3KO) and WT mice. It should be noted that a significant number of newborn O1/3dKO pups die shortly after birth, which could be attributed to their reduced ability to obtain food and water due to their reduced coordination and neuromuscular status (our observations and (11)). Only a small proportion of the O1/3dKO mice survive beyond 10 weeks of age. Hence, in our studies we used 6 to 8 week-old mice.

**Figure 2:**
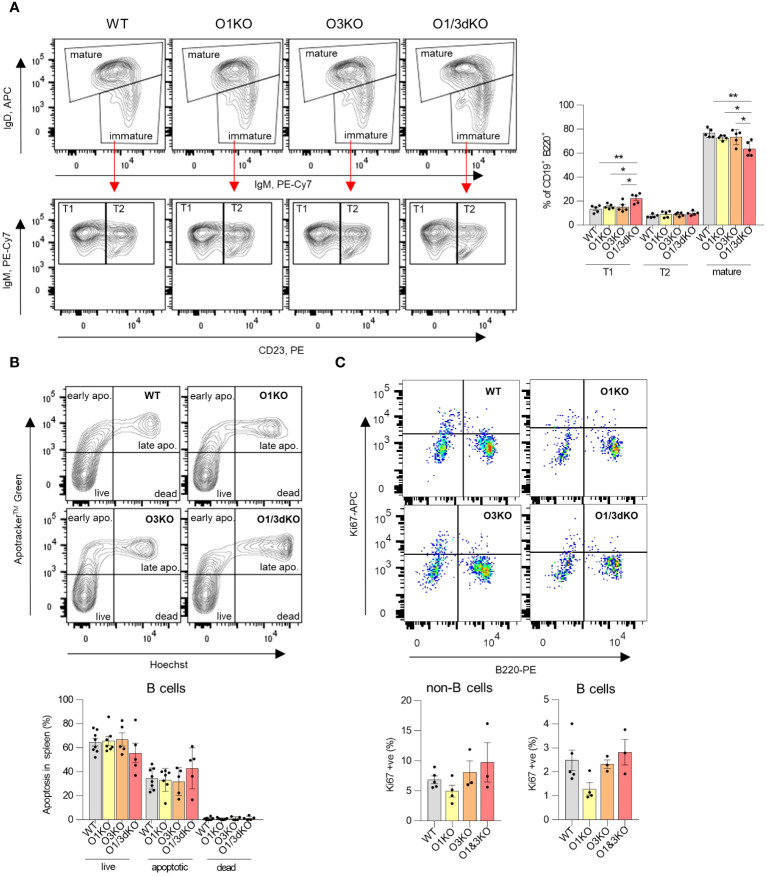
Development, apoptosis and proliferation of splenic B cells in the absence of different ORMDL proteins. **(A)** Viable single B220^+^ cells isolated from spleens were gated for IgM and IgD to distinguish between mature (IgD^high^) and immature (IgM^high^/IgD^low^) B cells (above). Immature B cells (below) were further analyzed for CD23 expression to distinguish between T1 (IgM^high^/CD23^-^) and T2 (IgM^high^/CD23^+^) transitional B cells. Percentage of different developmental stages of B cells were counted from all CD19 ^+^ B220^+^ cells. **(B)** Apoptosis of splenic B cells was assessed by double Apotracker™ Green/Hoechst staining, allowing to distinguish between live (Apotracker^-^/\Hoechst^-^), early apoptotic (Apotracker^+^/Hoechst^-^), late apoptotic (Apotracker^+^/Hoechst^+^) and dead cells (Apotracker^-^/Hoechst^+^). Representative image and percentage of individual B cell populations (n=5). **(C)** Proliferation of splenic B cells was measured using anti-Ki67 antibody. Single viable cells were stained with anti-B220 antibody to distinguish B and non-B cells (n=3). Data were analyzed by one-way ANOVA with Tukey´s post-test. (*P < 0.05, **P < 0.01). Each dot represents individual biological replicates. WT, *Ormdl1^-/-^* (O1KO), *Ormdl3^-/-^* (O3KO), *Ormdl1^-/-^/Ormdl3^-/-^* (O1/3dKO).

Detailed analysis of individual immune cell populations showed that all cell types, B cells, T cells, as well as myeloid cells were reduced in the spleens of O1/3dKO animals (**Figure 1D**). Further analysis revealed that the ratio between B cells and myeloid cells was slightly but significantly shifted in favor of myeloid cells in O1/3dKO mice (**Figure 1E**). However, despite the highly reduced immune cell content, the spleen architecture of O1/3dKO animals remained relatively normal (**Figure 1F**). B cells are crucially involved in the pathogenesis of multiple autoimmune diseases (41). Given the diminished B cell numbers in the spleen, we analyzed which developmental stages are most affected using detailed analysis of B cells in the spleen. Detailed analysis of splenic B cells revealed that all expected populations of B cells were present. Flow cytometry analysis showed that the number of mature B cells (IgD^+^) was significantly reduced at the expense of the immature transitional T1 (IgD^-^, IgM^+^, CD23^-^) population in spleens of O1/3dKO mice (**Figure 2A**).

As we observed reduced numbers of B cells in the spleens of O1/3dKO mice, we decided to test whether this was the result of increased apoptosis. We measured the proportion of viable, early apoptotic, late apoptotic and dead B cells in the spleens of both single and O1/3dKO mice. We did not observe any significant changes in the proportion of viable or apoptotic B cells between various genotypes (**Figure 2B**). In addition, the proliferation of splenic B cells was not affected by the loss of ORMDL proteins (**Figure 2C**).

Our results showed that the simultaneous deletion of ORMDL1 and ORMDL3 proteins leads to a significant decrease in the number of immune cells, particularly B cells, in the spleen. Although proliferation and apoptosis remained unchanged, B cell maturation was compromised in the spleens of O1/3dKO mice.

### 3.2 Blood homeostasis of O1/3dKO mice is impaired

**Figure 3:**
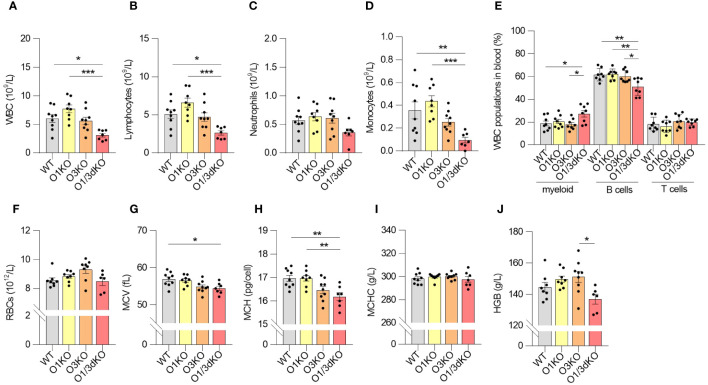
Blood homeostasis is altered in O1/3dKO mice. Blood samples collected from 6 to 8 week-old female mice were analyzed by Hemavet veterinary analyzer **(A–D, F–J)** or by flow cytometry **(E)**. **(A)** Total amount of white blood cells (WBC), and individual lymphocyte **(B)**, neutrophil **(C)** and monocyte **(D)** populations measured in peripheral blood. **(E)** Percentage of myeloid, T and B cells in blood. Single viable blood cells were stained with labeled antibodies against CD3, B220, CD11b and Gr-1 to distinguish individual lymphocyte populations. **(F)** Amount of red blood cells (RBC), **(G)** mean corpuscular volume (MCV), **(H)** mean corpuscular hemoglobin (MCH), **(I)** mean corpuscular hemoglobin concentration (MCHC), and **(J)** concentration of hemoglobin (HGB) in the blood of 6 to 8 week-old female mice (n ≥ 5). Data were analyzed by one-way ANOVA with Tukey´s post-test. (*P < 0.05, **P < 0.01, ***P < 0.001). Each dot represents individual biological replicates. WT, *Ormdl1^-/-^* (O1KO), *Ormdl3^-/-^* (O3KO), *Ormdl1^-/-^/Ormdl3^-/-^* (O1/3dKO).

Given the reduced number of immune cells in the spleen, we investigated whether their circulation was impaired. We measured the amount of white blood cells (WBC) and red blood cells (RBC) in blood using Hemavet veterinary hematological analyzer and flow cytometry. The amount of WBC, particularly lymphocytes and monocytes, was decreased in the blood of O1/3dKO mice (**Figures 3A–D**). In the blood, like in the spleen, the ratio of B cells to myeloid cells was slightly but significantly shifted in favor of myeloid cells in O1/3dKO mice (**Figure 3E**). The amount of RBC did not vary between the different genotypes in female mice (**Figure 3F**). We noted a reduction in RBC count in male O3KO mice (**Supplementary Figure 2A**). RBCs and RBC parameters from male and female mice were purposefully analyzed separately as differences in red cell mass and hemoglobin concentrations vary between males and females. In a physiological steady state, venous hemoglobin levels are lower in female mammals than males (42).

Analysis of RBC parameters revealed that the average volume of RBCs sampled from the blood of O1/3dKO mice was lower than that of WT mice, as reflected by the lower mean corpuscular volume (MCV) values (**Figure 3G**, **Supplementary Figure 2B**). As MCV values are an indication of the size of RBCs, we observed that the RBCs sampled from the blood of O1/3dKO mice were smaller than those of WT mice. This microcytosis, or smaller RBC size, was observed in both, male and female, O1/3dKO mice (**Figure 3G**, **Supplementary Figure 2B**). Consistent with the lower MCV values, the average mass of hemoglobin per RBC, or mean corpuscular hemoglobin (MCH), was decreased in the RBCs of O1/3dKO mice compared with the MCH of WT mice (**Figure 3H**, **Supplementary Figure 2C**). The concentrations of hemoglobin per RBC, or mean corpuscular hemoglobin concentration (MCHC), was comparable between the various genotypes (**Figure 3I**, **Supplementary Figure 2D**). Though we noted a trend toward reduced overall hemoglobin levels in female O1/3dKO mice, total hemoglobin concentrations (HGB) were significantly decreased in male mice only (**Figure 3J**, **Supplementary Figure 2E**). Overall, our results showed that blood homeostasis is significantly impaired in the concurrent absence of ORMDL1 and ORMDL3 proteins.

### 3.3 The BM of O1/3dKO mice is deficient in mature B cells

**Figure 4:**
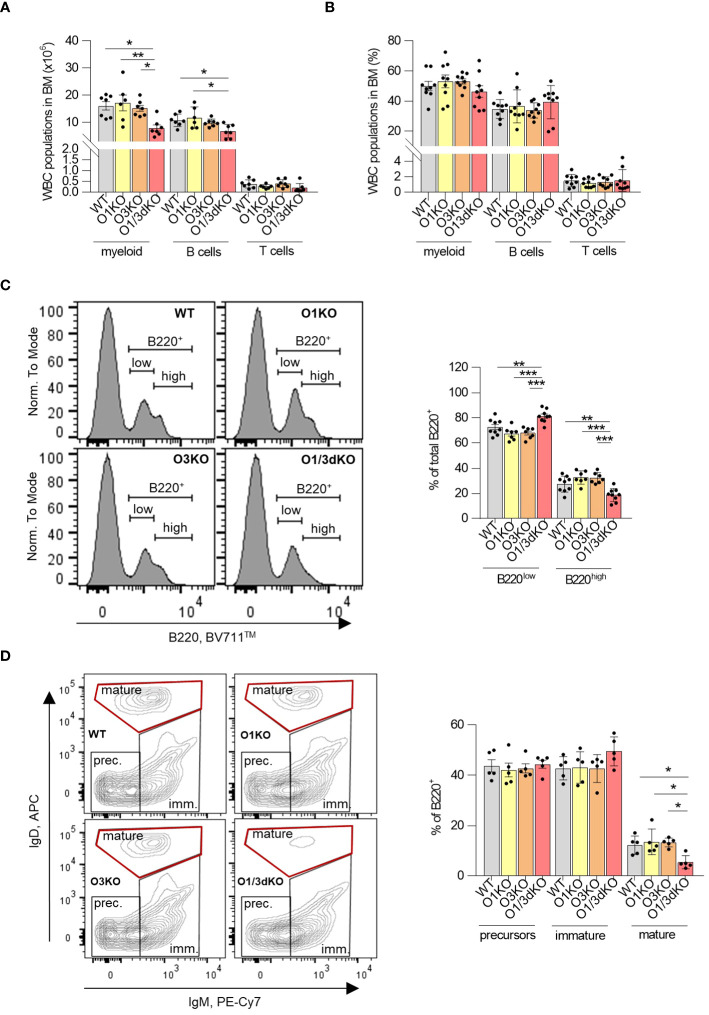
Normal development but decreased infiltration of mature B cells into BM of O1/3dKO mice. Cells isolated from BM of 6 to 8 week-old mice were analyzed by flow cytometry. Amount **(A)** and percentage **(B)** of myeloid, T and B cells in BM. Single viable BM cells were stained with labeled antibodies against CD3, B220, CD11b and Gr-1 to distinguish individual WBC populations. **(C)** Single viable BM cells were stained with anti-B220 antibody, representative image and percentage of B220^low^ and B220^high^ cells. **(D)** Single viable B220^+^ cells were gated for IgM and IgD expression, representative image and percentage of precursors (IgM^-^/IgD^-^), immature (IgM^+^/IgD^-/low^) and mature (IgM^-/low^/IgD^high^) B cells. Data were analyzed by one-way ANOVA with Tukey´s post-test. (*P < 0.05, **P < 0.01 and ***P < 0.001). Each dot represents independent biological replicate. WT, *Ormdl1^-/-^* (O1KO), *Ormdl3^-/-^* (O3KO), *Ormdl1^-/-^/Ormdl3^-/-^* (O1/3dKO).

Similarly to the spleen and peripheral blood, we found a reduced number of B cells in the BM of O1/3dKO animals as well (**Figure 4A**). However, this decrease of B cell numbers could be caused by the reduced size of O1/3dKO mice. The proportion of B cells within the WBC population in the BM remained unchanged (**Figure 4B**). To find out whether early development of B cells in the BM was affected by the deletion of ORMDL1 and ORMDL3 proteins, we analyzed the different developmental stages of B cells in the BM (**Figures 4C, D**). We found an alteration of B cell development in O1/3dKO BM, where the ratio of B220^high^ to B220^low^ cells was significantly shifted toward less mature B220^low^ cells (**Figure 4C**). In depth analysis of these populations showed reduced numbers of mature B cells (IgD^+^, IgM^-^) in O1/3dKO mice while no significant changes in the numbers of B cell precursors (IgD^-^, IgM^-^) and immature B cells (IgD^-^, IgM^+^) were observed (**Figure 4D**). Since the presence of mature B cells in the BM is dictated by the circulation of mature B cells from secondary lymphoid organs and peripheral blood, it is likely that the reduced number of mature B cells in the BM of O1/3dKO mice is a consequence of the reduced number of mature cells found in their blood and spleen (**Figures 1D**, **3E**). As such, it appears that B cell development was not disrupted in the BM of O1/3dKO mice. Deletion of SPT long-chain base subunit 1 (SPTLC1), activity of which is controlled by ORMDL proteins, resulted in compromised myelopoiesis, characterized by diminished numbers of common myeloid progenitors (CMPs) and granulocyte-macrophage progenitors (GMPs), although the proportion of hematopoietic stem cells was increased (17). To find out whether early development of lymphocytes is affected by the loss of ORMDL proteins, we studied the proportion of common lymphoid progenitors (CLPs), CMPs and GMPs, as well as megakaryocyte/erythrocyte progenitors (MEPs) in BM. We did not find any significant differences in the number of different immune cell progenitors between the various genotypes (**Supplementary Figure 3**). Our data show that the deletion of ORMDL proteins does not affect hematopoietic progenitor distributions in the BM.

### 3.4 Lymphocyte homing to the spleen and BM is not affected by the loss of ORMDL1 and ORMDL3 proteins

**Figure 5:**
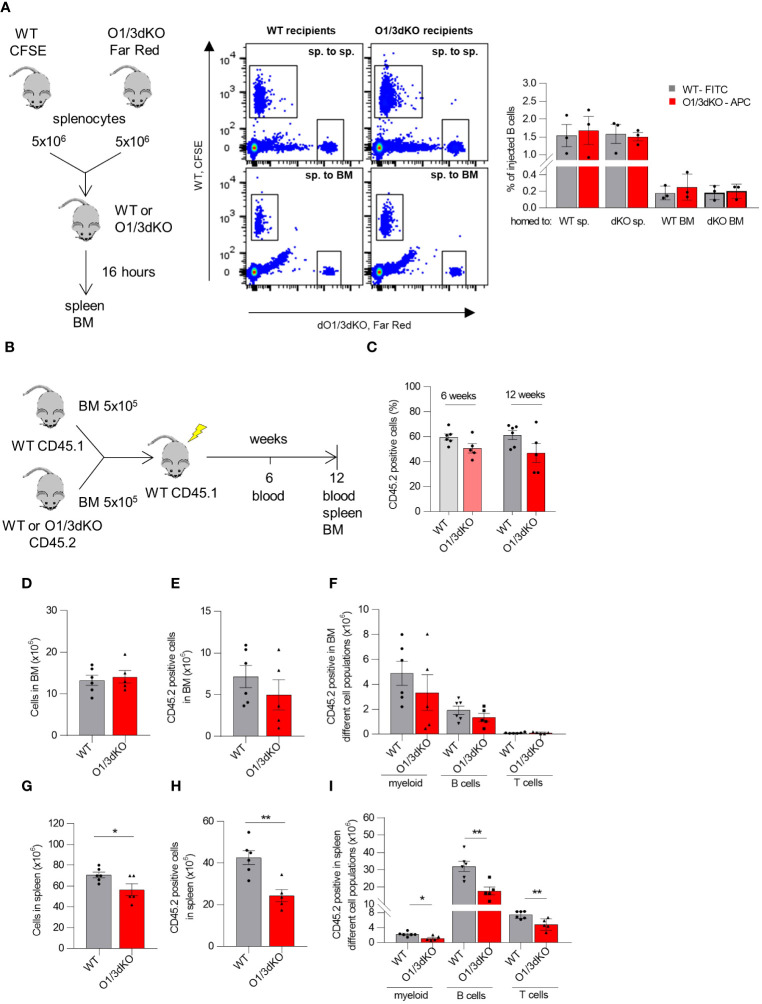
Homing and competitive transplantation assay of lymphocytes with deleted ORMDL1 and ORMDL3 proteins. **(A)** Cell Trace™-CFSE-labeled WT and Cell Trace™-Far Red-labeled O1/3dKO splenocytes were injected via tail vein in 1:1 ratio into WT or O1/3dKO recipient mice. After 16 hours, the mice were sacrificed, their organs were harvested and cells were analyzed by flow cytometry. Schema of experimental design, representative flow cytometry gating comparing homing of WT and O1/3dKO splenic cells into the spleen (sp. to sp.), or BM (sp. to BM), and percentage of homed B cells. **(B–I)** WT BM CD45.1 cells were mixed with BM CD45.2 cells isolated from WT or O1/3dKO mice in 1:1 ratio and transplanted into lethally irradiated CD45.1 WT recipient mice. **(B)** Schematic representation of the experimental setup. **(C)** Percentage of CD45.2^+^ cells derived from WT or O1/3dKO BM cells in the blood of recipient animals 6 or 12 weeks post transplantation. Number of all cells **(D)**, CD45.2^+^ cells **(E)**, and amount of different cell types in BM **(F)** of recipient animals. Number of all cells **(G)**, CD45.2^+^ cells **(H)** and amount of different cell types in the spleen **(I)** of recipient animals. Data were analyzed by one-way ANOVA with Tukey´s post-test. *P < 0.05 **P < 0.01). Each dot represents independent biological replicates. WT, *Ormdl1^-/-^* (O1KO), *Ormdl3^-/-^* (O3KO), *Ormdl1^-/-^/Ormdl3^-/-^* (O1/3dKO).

Given the markedly reduced cellularity of the spleen of O1/3dKO mice in contrast to only limited reduction of immune cell numbers in the blood and BM, we reasoned that a defect in lymphocyte recirculation may underlie the observed phenotype. We therefore evaluated whether the simultaneous deletion of ORMDL1 and ORMDL3 affected B cell homing to the spleen and BM. Lymphocytes isolated from the spleens of WT and O1/3dKO mice were labeled with Cell Tracers (CFSE and CFSE-Far Red, respectively), injected into WT or O1/3dKO mice and allowed to migrate to the recipients’ organs for 16 hours (**Figure 5A**). Flow cytometry was used to identify the relative frequencies of adoptively transferred lymphocytes and to distinguish B cells. The obtained data showed that adoptively transferred splenocytes from both genotypes were able to home to the spleen and, with less efficiency, to the BM. However, no significant differences in homing were observed between WT and O1/3dKO donors and recipients. Our data revealed that the significant reduction of B cells in the spleen of O1/3dKO mice is not caused by impaired homing.

### 3.5 The reduced number of B cells in the spleens of O1/3dKO mice is partly dependent on lymphocyte intrinsic factors

Members of the ORMDL family are expressed not only in immune cells but also in other cell types (5). It has been shown that under pathological conditions, the degree of expression of ORMDL proteins is cell-type specific. For example, upon allergen challenge, ORMDL3 expression was increased more than 100 times in lung epithelial cells and macrophages, but remained unchanged in blood neutrophils (22). To clarify whether the observed defects in B cell development and homeostasis were caused by cell intrinsic or extrinsic factors, we performed a competitive BM transplantation assay. BM cells isolated from WT or O1/3dKO mice were transplanted into lethally irradiated congenic mice together with support WT congenic BM cells in a 1:1 ratio (**Figure 5B**). To assess short term hematopoietic reconstitution, recipient blood was analyzed 6 weeks after transplantation for the presence of newly developed lymphocytes. We did not observe a difference in the percentage of total CD45.2+ cells reconstituted from WT and O1/3dKO donors in the blood of recipient mice 6 weeks post transplantation (**Figure 5C**). Long term repopulation abilities were analyzed 12 weeks post transplantation. In the blood, similarly to the result observed 6 weeks post transplantation, no differences in total CD45.2+ cells reconstituted from WT and O1/3dKO donors were noted (**Figure 5C**). Comparable results were noted in the BM, where no significant changes in CD45.2+ engraftment between WT and O1/3dKO cells were observed (**Figures 5D, E**). In addition, no differences in the engraftment of individual WT and O1/3dKO myeloid cells or lymphocytes were detected (**Figure 5F**). In the spleen, however, we noted a decreased number of splenocytes in mice transplanted with cells isolated from the BM of O1/3dKO mice compared with cells from WT donors (**Figure 5G**). The deletion of ORMDL1 and ORMDL3 proteins affected the overall engraftment of CD45.2+ cells in the spleen, as well as individual immune cell types, myeloid, B cells and T cells, equally (**Figures 5H, I**). From these experiments we conclude that the development of O1/3dKO lymphocytes in the spleen is, at least partially, dependent on intrinsic factors inherent to hematopoietic cells.

### 3.6 Deletion of different ORMDL proteins specifically affects sphingolipid synthesis in different organs

**Figure 6:**
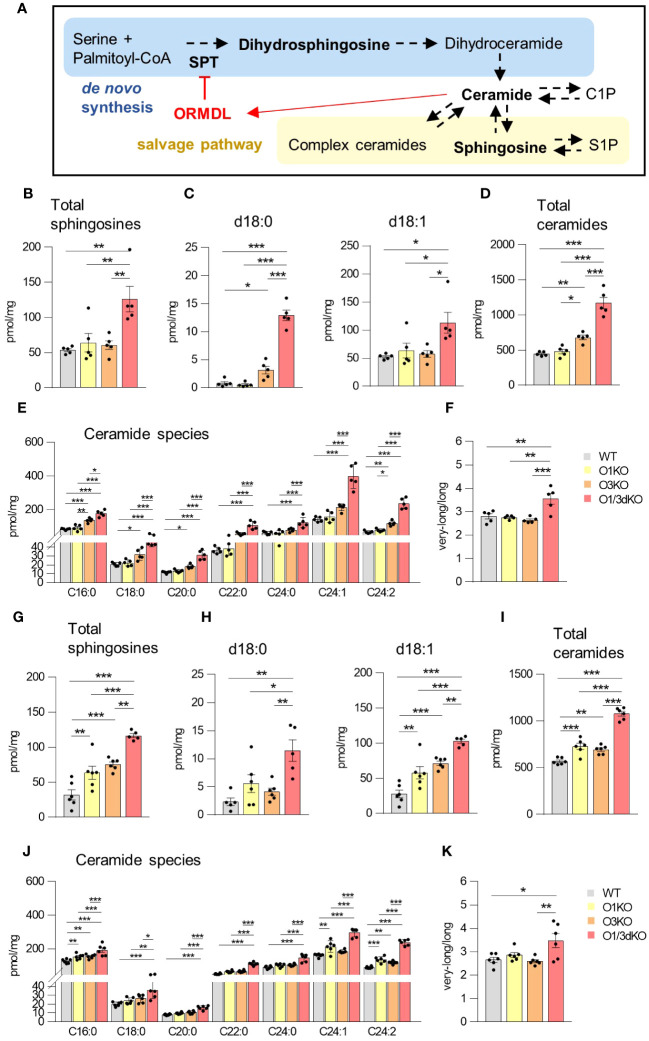
Increased sphingolipid production in the spleen and BM of O1/3dKO mice. **(A)** Schema of *de novo* sphingolipid biosynthesis (blue box) and sphingolipid salvage pathway (beige box) with the feedback inhibition by ORMDL proteins through sensing of elevated ceramide levels (red). SPT – serine palmitoyltransferase, CoA – coenzyme **(A)** Measured sphingolipids are in bold. LC-ESI-MS/MS analysis of sphingolipids in spleens **(B–F)** and BMs **(G–K)** of 6 to 8 week-old WT and O1/3dKO mice. **(B, G)** The sum of total sphingosines (d18:1, d18:0, d20:1, and d20:0). **(C, H)** Concentration of dihydrosphingosine (d18:0) and sphingosine (d18:1). **(D, I)** The sum of total ceramide derived from d18:1 sphingosine. **(E, J)** Levels of individual ceramide species with different fatty acid chains. **(F, K)** Ratio of very-long (C22-C26) vs. long (C14-C20) acyl chain ceramide molecular species. Data were analyzed by one-way ANOVA with Tukey´s post-test. (*P < 0.05, **P < 0.01 and ***P < 0.001). Each dot represents independent biological replicate. WT, *Ormdl1^-/-^* (O1KO), *Ormdl3^-/-^* (O3KO), *Ormdl1^-/-^/Ormdl3^-/-^* (O1/3dKO).

Sphingolipids are able to regulate the migration of several cell types, including lymphocytes (43). ORMDL proteins are important regulators of sphingolipid biosynthesis (3–5). Among individual ORMDL proteins, deletion of ORMDL3 causes the highest elevation in sphingolipid levels, which can be further potentiated by the deletion of other family members, depending on the cell type (11–13, 19). To define the role of ORMDL proteins in sphingolipid synthesis in the BM and spleen, we extracted sphingolipids from BM cells and splenocytes obtained from WT, O1KO, O3KO and O1/3dKO mice and analyzed them by tandem mass spectrometry. We measured levels of dihydrosphingosine (d18:0), generated exclusively as an early intermediate in *de novo* sphingolipid biosynthesis, as well as sphingosine (d18:1) and different ceramides generated by both *de novo* and salvage sphingolipid biosynthesis pathways (**Figure 6A**). Simultaneous deletion of ORMDL1 and ORMDL3 proteins led to the greatest increase in the levels of all sphingolipids measured. This combined deletion influenced both *de novo* and recycling pathways of sphingolipid biosynthesis in the spleen and BM (**Figures 6B–K**). The effect of individual ORMDL family members on the synthesis of different sphingolipid subtypes was distinct between the spleen and BM. In the spleen, the deletion of ORMDL3 caused an increase in *de novo* sphingolipid synthesis, as was evident from the measured concentration of dihydrosphingosine (d18:0) (**Figure 6C**). The amount of sphingosine (d18:1), which is produced by salvage pathways, was not affected (**Figure 6C**). Deletion of ORMDL3 also led to an increased production of ceramides in the spleen (**Figures 6D, E**). The increase in ceramides was primarily noted among molecular species with long (C16-C20) saturated fatty acid chains (**Figures 6E, F**). In the BM, single deletion of ORMDL1 led to an increase in total sphingosines and ceramides comparable to the changes observed with the single deletion of ORMDL3 (**Figures 6G, I**). In contrast to the spleen, the increase in sphingolipids was mainly driven by the salvage pathway, as the amount of *de novo* intermediate product, dihydrosphingosine, was not significantly altered in single KO cells (**Figure 6H**). It should also be noted that synthesis of ceramides with very-long fatty acid chains (C22-C24) in both, the spleen and BM, was mostly affected by the simultaneous loss of ORMDL1 and ORMDL3 proteins (**Figures 6E, J**). This was reflected by an increased ratio of synthetized ceramides with very-long vs. long acyl chains (**Figures 6D, K**). These data confirm that the influence of individual ORMDL family members on the production of different sphingolipid species is cell-type dependent. The simultaneous deletion of ORMDL1 and ORMDL3 augments the effects seen with the loss of individual ORMDL proteins.

## 4 Discussion

Dysregulation of sphingolipid synthesis leads to many human diseases, where increased, as well as decreased, sphingolipid production is critical for disease development and manifestation (15, 43–45). ORMDL proteins are essential regulators of sphingolipid production by ceramide-sensitive inhibition of the SPT enzyme (3, 8, 9, 14). In the current study, we analyzed the immune cells of mice in which one or two *Ormdl* genes were deleted. Deletion of all three *Ormdl* genes simultaneously is incompatible with life (11, 12). We found that mice with simultaneous deletions of ORMDL1 and ORMDL3 proteins had reduced spleen sizes with diminished immune cell content. These mice had fewer circulating lymphocytes, particularly mature B cells, and suffered from slight anemia. The altered development of O1/3dKO splenocytes partially depended on impaired intrinsic properties of hematopoietic stem cells. Surprisingly, the homing of lymphocytes into the BM and spleen was not affected by the loss of ORMDL proteins. No significant changes in the number of splenocytes was observed in mice with single deletions of ORMDL proteins nor double KO mice with a concurrent deletion of ORMDL2.

It has previously been shown that the deletion of ORMDL3 leads to the decreased survival of splenic B cells by increased apoptosis and decreased autophagy (20). In the present study, we only observed a small but insignificant decrease in the number of B cells in O3KO mice. This discrepancy could be caused by different techniques used to prepare the KO mice or by the stringency of statistical analysis. We compared four different genotypes with each other, whereas Dang et al. (20) compared just O3KO to WT. In our study, more pronounced changes observed in the immune cell content of O1/3dKO mice manifested as statistically significant, while smaller differences between genotypes were not highlighted by the statistical comparisons used. Due to the frailty and increased mortality of O1/3dKO mice, we performed experiments using mice that were 6 to 8 weeks of age, which may have also contributed to the differences between the results found in our work and the previous study (20). Despite their decreased number in the spleen, we did not observe a significant increase in apoptosis of O1/3dKO splenic B cells, as was detected in O3KO B cells by Dang et al. (20).

ORMDL proteins are highly conserved, and the homology between paralogs on amino acid levels is more than 80% (5). In our previous work, we observed functional redundancy of ORMDL1 and ORMDL2 proteins in regulating sphingolipid production in mast cells (12, 13). Deletion of these two members of the ORMDL family, separately or simultaneously, did not affect sphingolipid production. However, in the absence of ORMDL3, which alone increased sphingolipid levels, the additional deletion of any other ORMDL family member led to a multifold increase in sphingolipid production (12, 13). In murine brains, the sole deletion of ORMDL3 led to increased sphingolipid production. The effect was multiplied by the concurrent deletion of ORMDL1. In contrast, the simultaneous deletion of ORMDL2 and ORMDL3 kept sphingolipid production at WT levels (11). In this study, we observed that, while reduction of ORMDL proteins in splenocytes led to similar results to those observed in mast cells, in immune cells isolated from the BM, even a single deletion of ORMDL1 led to increased sphingolipid production to a similar extent that was observed in the absence of ORMDL3 protein. Recent data showed that ORMDL3 is the most potent ORMDL paralog in sensing C6-ceramide, which consequently triggered ORMDL-dependent SPT suppression. Complexes of SPT with ORMDL1 or ORMDL2 were inhibited by C6-ceramide binding by only 30% and 10%, respectively (9). It should be noted that the study of Xie et al. (9) used the binding of C6-ceramide to model SPT inhibition by ORMDL proteins. However, a variety of different ceramide species with different chain lengths are present in cells *in vivo*. Expression of individual ORMDL family members differs slightly between different tissues and cell types (Human atlas). Profiles of sphingolipid species are also cell-type specific (14, 19, 46). These factors may play an important role in the different sensitivity of particular cell types to the deletion of various ORMDL family members. As already mentioned, all three proteins of the ORMDL family are highly homologous, with 80% amino acid identity among human paralogs and 95% identity with their murine orthologues (5). The deletion of one or two members of the ORMDL family during embryonic development may trigger compensatory mechanisms that prevent damage to the organism. We have previously shown that reduction of ORMDL3 in mature mast cells affected their proinflammatory phenotype to a greater extent than was observed in mast cells isolated from whole-body O3KO mice (12). Our results indicated that the function of individual members of the ORMDL family can partially compensate each other during development.

One of the important drivers of lymphocyte circulation is a gradient of S1P that is formed following the phosphorylation of sphingosine by sphingosine kinases (47–49). We hypothesized that the reduced number of immune cells in the spleen might be due to their poor homing into the target organ. However, our experiments demonstrated that the absence of ORMDL1 and ORMDL3 proteins in adoptively transferred lymphocytes and/or in recipient animals did not affect homing efficiency. Levels of S1P in blood and tissues are tightly controlled (50). S1P can be dephosphorylated back to sphingosine or irreversibly cleaved by S1P lyase (51). This process is highly active in most tissues with the exception of erythrocytes and platelets due to decreased S1P phosphatase activity and the absence of S1P lyase (51). A sharp gradient of S1P concentration between blood and lymph on the one hand, and lymphoid tissues on the other hand, drives lymphocyte egress from lymphoid tissues into the circulation. This process is controlled by membrane expression of S1PR1 that is internalized upon S1P binding (47–49). We previously detected elevated S1P levels in the serum of O3KO and O2/3dKO mice (13). Nevertheless, here we show that these mice have normal amounts of splenocytes. Since we detected an increased production of sphingolipids in both, the spleens and BM of O1/3dKO mice, it is likely that the sphingolipid gradient was not disrupted, but levels of individual sphingolipids were proportionally increased. It should also be noted that increased S1P levels may contribute to the stabilization of ORMDL proteins through S1P-S1PR1 and thus contribute to maintaining sphingolipid homeostasis by a negative feedback loop (10).

Only limited research addressed the role of sphingolipids in B cell differentiation. Our competitive transplantation assay revealed that the deletion of ORMDL1 and ORMDL3 proteins in hematopoietic stem cells leads to reduced numbers of immune cells in the spleen. Sphingolipid homeostasis is important for proper hematopoiesis, and different sphingolipid profiles were identified across the human hematopoietic hierarchy (46). S1P bound to high-density lipoproteins restrains lymphocyte development (52). Diminished sphingolipid synthesis upon deletion of SPT subunits, SPTLC1 and ssSPTa, leads to defective myeloid differentiation (17, 18). However, our present data show that deleting sphingolipid synthesis regulators, ORMDL1 and ORMDL3, did not significantly affect early hematopoietic stem cell differentiation.

Though we did not detect defects in early B cell development in the BM of O1/3dKO mice, we did note a high population of immature B cells. B cells are involved in the development of autoimmune diseases through their interaction with T lymphocytes and their potential to produce autoantibodies (41). Immature transitional B cells actively exert immunomodulatory functions through the production of cytokines, thereby influencing both, T cell responses and the fate of autoreactive B cells (53). Abnormal distributions of various transitional B cell subsets have been identified in patients with autoimmune diseases (54). Patients with SLE have been found to have increased numbers of circulating T1 B cells (55). In our study, we observed elevated numbers of T1 B cells in the spleens of O1/3dKO mice, a characteristic, which may potentially predispose to autoimmune diseases.

Regulatory mechanisms, or checkpoints, which eliminate autoreactive B cells occur in the BM and spleen. Immature B cells that fail to develop tolerance at these checkpoints have been implicated in the pathogenesis of many autoimmune diseases, such as RA, T1D and SLE (56). Tolerance checkpoints rely on receptor editing, anergy and clonal deletion in removing self-reactive B cells, which are in turn dependent on robust B cell receptor (BCR) signaling. As such, defective BCR signaling may give rise to autoimmune diseases (41). Lipid rafts, which are enriched in sphingolipids, influence plasma membrane fluidity and protein-protein interactions (57). Defective recruitment of proteins to lipid rafts due to changes in the composition of lipid rafts on B cell plasma membranes leads to continuous BCR activation and production of autoantibodies (58, 59). O1/3dKO mice displayed increased production of sphingolipids in both, the BM and spleen, which may interfere with the proper development and removal of autoreactive B cells. These and the aforementioned findings may contribute to a better understanding of how ORMDL proteins are involved in the etiology of autoimmune diseases.

Studies have found that females are at a higher risk of autoimmune diseases than males. Female sex hormones influence the function and development of adaptive immune responses (60). The XX karyotype constitutes an additional risk for autoimmune diseases, independent of hormonal status. In particular, mechanisms associated with X chromosome inactivation, which are absent in males, trigger antigenic responses (61). Despite the increased risk of autoimmune diseases in females, we did not observe differences in B cell development between male and female mice in our current study. The changes in B cell maturation appear to occur independently of mechanisms associated with the increased risk of autoimmune diseases in females.

RBC counts and hemoglobin levels differed between male and female mice. In particular, male O3KO and O1/3dKO mice were significantly anemic with reduced HGB levels. Male and female sex hormones varyingly influence RBC and hemoglobin formation by stimulating or inhibiting erythropoietin production, respectively (62). Through their bioactive properties, sphingolipids have been shown to modulate steroid hormone synthesis (63). In particular, ceramides suppress androgen production (64). High concentrations of ceramides in male O3KO and O1/3dKO mice may potentially reduce androgen levels, thereby dampening the stimulatory effect that androgens have on erythropoietin, leading to decreased hemoglobin levels and anemia.

**Figure 7:**
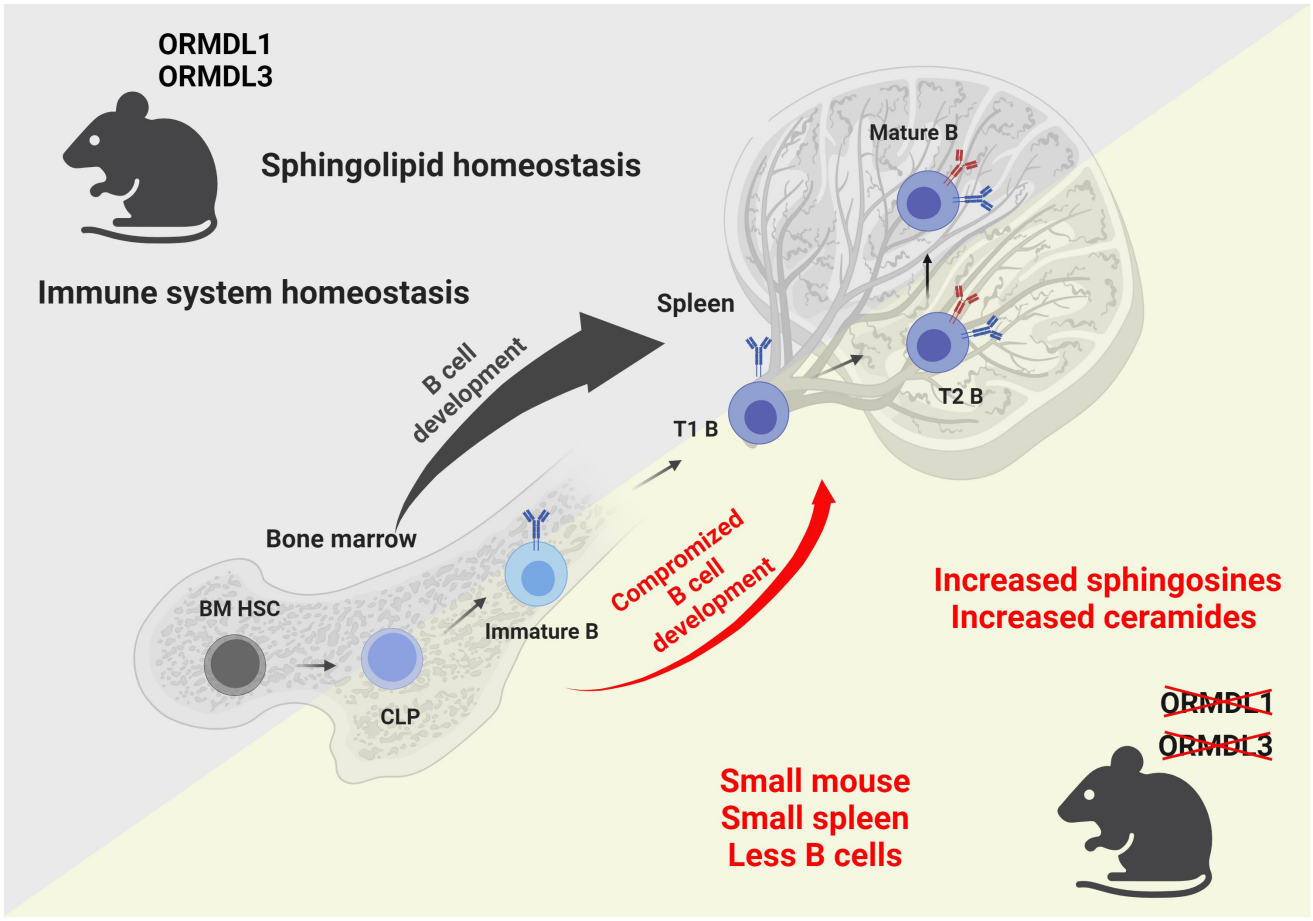
Graphical summary of the findings observed in mice with simultaneous deletions of ORMDL1 and ORMDL3 proteins. WT mice, depicted in the upper triangle, maintain sphingolipid homeostasis and normal B cell development within the bone marrow and spleen. Mice with double deletions of the sphingolipid biosynthesis regulators ORMDL1 and ORMDL3, depicted in the lower triangle, present with a significant pathological phenotype. O1/3dKO mice are smaller in stature. Furthermore, these mice have disproportionally smaller spleens with reduced splenocyte numbers and compromised B cell development that is characterized by increased proportions of T1 B cells in the spleen with decreased circulating mature B cells. Dysregulated sphingolipid biosynthesis leads to increased sphingosines and ceramides in both, the BM and spleens of O1/3dKO mice. BM HSC, bone marrow hematopoietic stem cells; CLP, common lymphoid progenitor cells; Immature B, Immature B cells; T1 B, Transitional 1 B cells; T2 B, Transitional 2 B cells; Mature B, Mature B cells. Created with BioRender.com.

Taken together, we show that the physiological expression of ORMDL proteins is essential for blood cell homeostasis, and the proper development and circulation of immune cells (**Figure 7**). We also prove that the influence of individual ORMDL family members on the production of different sphingolipid species is cell-type specific. B cell maturation is compromised in the spleens of O1/3dKO mice. Simultaneous deletion of ORMDL1 and ORMDL3 proteins disrupts immune cell circulation and homeostasis. We prove that the reduction of immune cells in O1/3dKO mice is not caused by aberrant homing but is at least partially dependent on lymphocyte intrinsic factors.

### 4.1 Limitations of the study

In this study, we analyzed the effects of deleting ORMDL proteins in immune cell development. Increased mortality of O1/3dKO mice shortly after birth and weaning precludes some *in vivo* experiments. Thus, it is not possible to demonstrate the role of an ORMDL1 and ORMDL3 deficient environment in immune cell development by transplantation assay. It is also challenging to study the function of the immune system of O1/3dKO mice *in vivo*, given their baseline health status. ORMDL family members are highly homologous and they are at least partially redundant. The degree of these compensatory mechanisms during development is highly variable. Thus, detailed understanding of the mechanism by which the deletion of individual ORMDL proteins affects the immune system requires further study.

## Data availability statement

The raw data supporting the conclusions of this article will be made available by the authors, without undue reservation.

## Ethics statement

The animal study was approved by ethical committee of the Institute of Molecular Genetics (permit number 12135/2010-17210) and Czech national guidelines (2048/2004-1020). The study was conducted in accordance with the local legislation and institutional requirements.

## Author contributions

LD: Conceptualization, Formal analysis, Investigation, Validation, Writing – original draft, Methodology. VB: Formal analysis, Investigation, Methodology, Writing – review & editing. MA: Formal analysis, Investigation, Methodology, Writing – review & editing. LK: Formal analysis, Funding acquisition, Investigation, Methodology, Writing – review & editing. SG: Formal analysis, Investigation, Methodology, Writing – review & editing. MA-J: Funding acquisition, Resources, Supervision, Writing – review & editing. PD: Conceptualization, Funding acquisition, Resources, Supervision, Writing – original draft. IH: Conceptualization, Formal analysis, Funding acquisition, Investigation, Methodology, Resources, Supervision, Writing – original draft.
