# Supplementary material for: Simultaneous deletion of ORMDL1 and ORMDL3 proteins disrupts immune cell homeostasis
Source: Front Immunol. 2024 Apr 23;15:1376629. doi: 10.3389/fimmu.2024.1376629 (PMC11074395; doi:10.3389/fimmu.2024.1376629)
Supplement: Supplementary file 1 [file Presentation_1.pptx]

## Slide 1
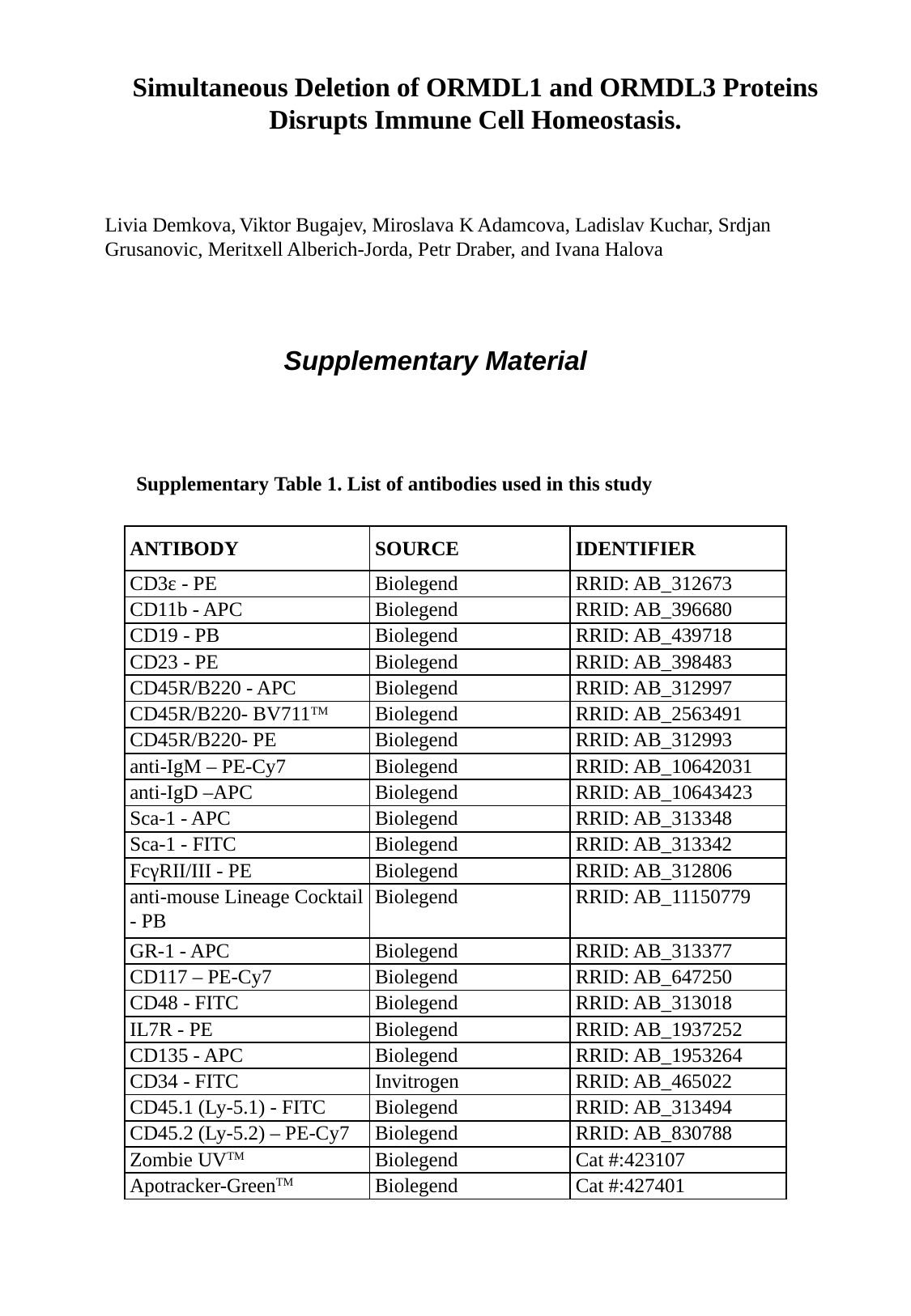

Simultaneous Deletion of ORMDL1 and ORMDL3 Proteins Disrupts Immune Cell Homeostasis.
Livia Demkova, Viktor Bugajev, Miroslava K Adamcova, Ladislav Kuchar, Srdjan Grusanovic, Meritxell Alberich-Jorda, Petr Draber, and Ivana Halova
Supplementary Material
Supplementary Table 1. List of antibodies used in this study
| ANTIBODY | SOURCE | IDENTIFIER |
| --- | --- | --- |
| CD3ε - PE | Biolegend | RRID: AB\_312673 |
| CD11b - APC | Biolegend | RRID: AB\_396680 |
| CD19 - PB | Biolegend | RRID: AB\_439718 |
| CD23 - PE | Biolegend | RRID: AB\_398483 |
| CD45R/B220 - APC | Biolegend | RRID: AB\_312997 |
| CD45R/B220- BV711TM | Biolegend | RRID: AB\_2563491 |
| CD45R/B220- PE | Biolegend | RRID: AB\_312993 |
| anti-IgM – PE-Cy7 | Biolegend | RRID: AB\_10642031 |
| anti-IgD –APC | Biolegend | RRID: AB\_10643423 |
| Sca-1 - APC | Biolegend | RRID: AB\_313348 |
| Sca-1 - FITC | Biolegend | RRID: AB\_313342 |
| FcγRII/III - PE | Biolegend | RRID: AB\_312806 |
| anti-mouse Lineage Cocktail - PB | Biolegend | RRID: AB\_11150779 |
| GR-1 - APC | Biolegend | RRID: AB\_313377 |
| CD117 – PE-Cy7 | Biolegend | RRID: AB\_647250 |
| CD48 - FITC | Biolegend | RRID: AB\_313018 |
| IL7R - PE | Biolegend | RRID: AB\_1937252 |
| CD135 - APC | Biolegend | RRID: AB\_1953264 |
| CD34 - FITC | Invitrogen | RRID: AB\_465022 |
| CD45.1 (Ly-5.1) - FITC | Biolegend | RRID: AB\_313494 |
| CD45.2 (Ly-5.2) – PE-Cy7 | Biolegend | RRID: AB\_830788 |
| Zombie UVTM | Biolegend | Cat #:423107 |
| Apotracker-GreenTM | Biolegend | Cat #:427401 |

## Slide 2
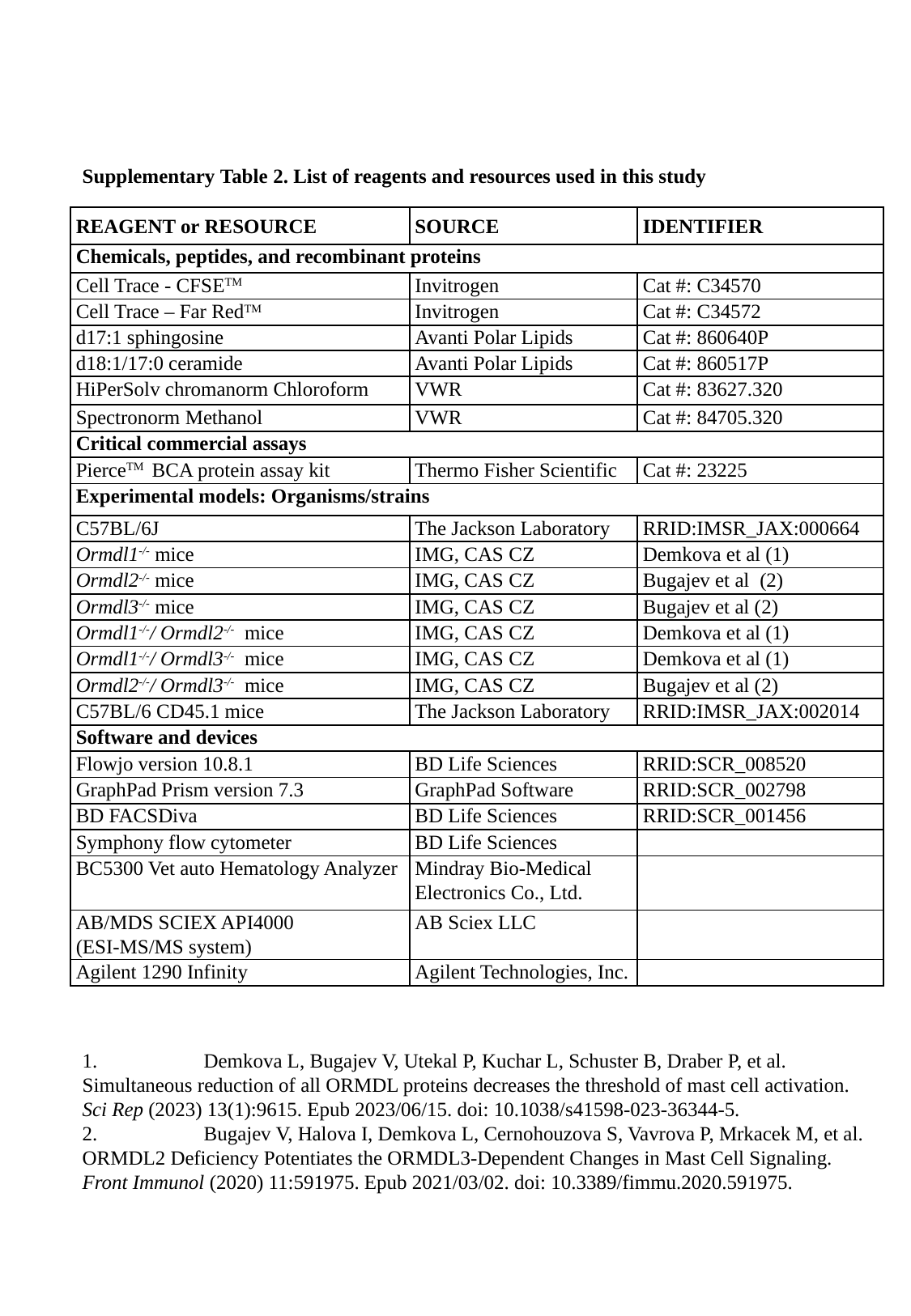

Supplementary Table 2. List of reagents and resources used in this study
| REAGENT or RESOURCE | SOURCE | IDENTIFIER |
| --- | --- | --- |
| Chemicals, peptides, and recombinant proteins | | |
| Cell Trace - CFSETM | Invitrogen | Cat #: C34570 |
| Cell Trace – Far RedTM | Invitrogen | Cat #: C34572 |
| d17:1 sphingosine | Avanti Polar Lipids | Cat #: 860640P |
| d18:1/17:0 ceramide | Avanti Polar Lipids | Cat #: 860517P |
| HiPerSolv chromanorm Chloroform | VWR | Cat #: 83627.320 |
| Spectronorm Methanol | VWR | Cat #: 84705.320 |
| Critical commercial assays | | |
| PierceTM BCA protein assay kit | Thermo Fisher Scientific | Cat #: 23225 |
| Experimental models: Organisms/strains | | |
| C57BL/6J | The Jackson Laboratory | RRID:IMSR\_JAX:000664 |
| Ormdl1-/- mice | IMG, CAS CZ | Demkova et al (1) |
| Ormdl2-/- mice | IMG, CAS CZ | Bugajev et al (2) |
| Ormdl3-/- mice | IMG, CAS CZ | Bugajev et al (2) |
| Ormdl1-/-/ Ormdl2-/- mice | IMG, CAS CZ | Demkova et al (1) |
| Ormdl1-/-/ Ormdl3-/- mice | IMG, CAS CZ | Demkova et al (1) |
| Ormdl2-/-/ Ormdl3-/- mice | IMG, CAS CZ | Bugajev et al (2) |
| C57BL/6 CD45.1 mice | The Jackson Laboratory | RRID:IMSR\_JAX:002014 |
| Software and devices | | |
| Flowjo version 10.8.1 | BD Life Sciences | RRID:SCR\_008520 |
| GraphPad Prism version 7.3 | GraphPad Software | RRID:SCR\_002798 |
| BD FACSDiva | BD Life Sciences | RRID:SCR\_001456 |
| Symphony flow cytometer | BD Life Sciences | |
| BC5300 Vet auto Hematology Analyzer | Mindray Bio-Medical Electronics Co., Ltd. | |
| AB/MDS SCIEX API4000 (ESI-MS/MS system) | AB Sciex LLC | |
| Agilent 1290 Infinity | Agilent Technologies, Inc. | |
1.	Demkova L, Bugajev V, Utekal P, Kuchar L, Schuster B, Draber P, et al. Simultaneous reduction of all ORMDL proteins decreases the threshold of mast cell activation. Sci Rep (2023) 13(1):9615. Epub 2023/06/15. doi: 10.1038/s41598-023-36344-5.
2.	Bugajev V, Halova I, Demkova L, Cernohouzova S, Vavrova P, Mrkacek M, et al. ORMDL2 Deficiency Potentiates the ORMDL3-Dependent Changes in Mast Cell Signaling. Front Immunol (2020) 11:591975. Epub 2021/03/02. doi: 10.3389/fimmu.2020.591975.

## Slide 3
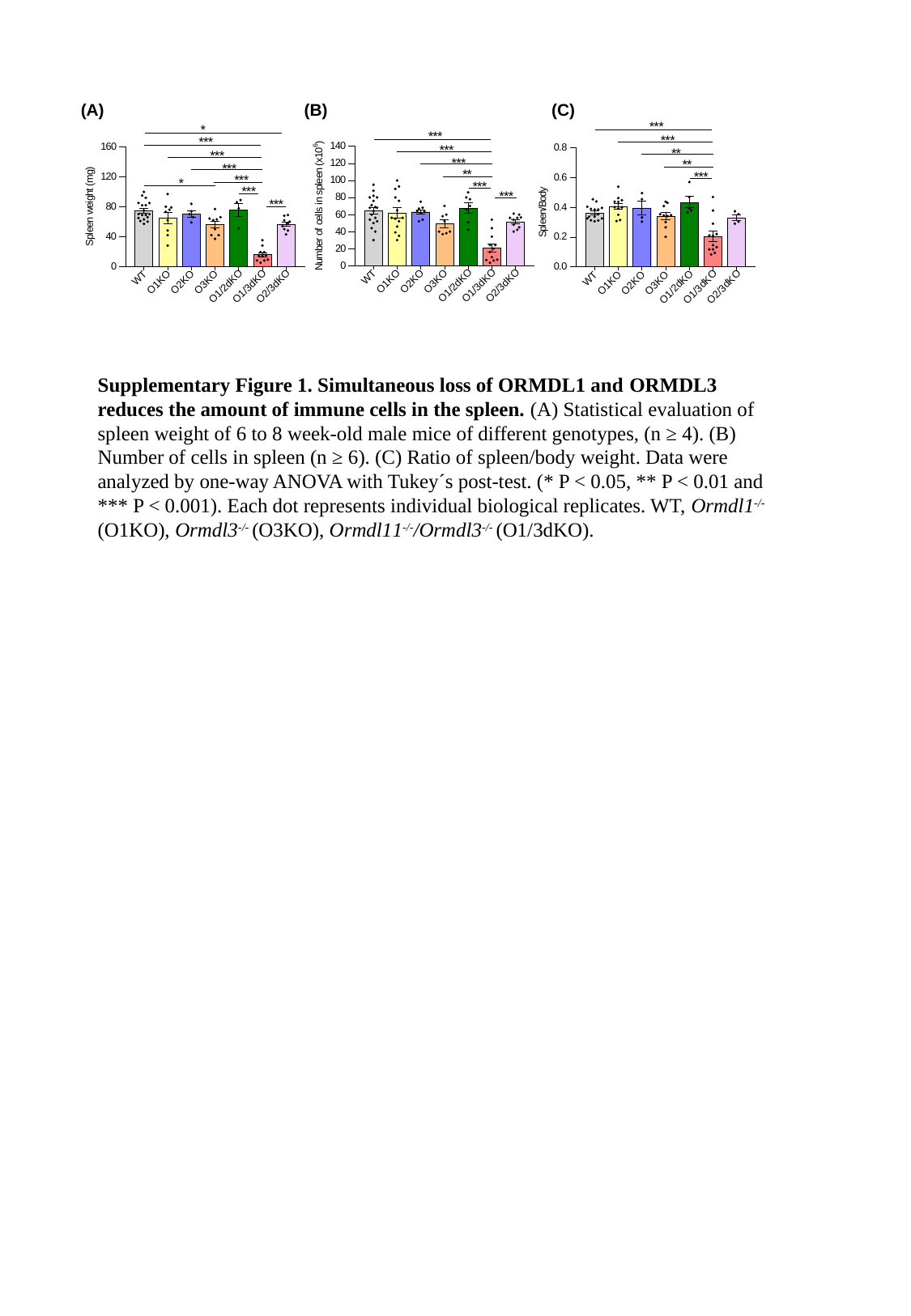

(A) (B) (C)
Supplementary Figure 1. Simultaneous loss of ORMDL1 and ORMDL3 reduces the amount of immune cells in the spleen. (A) Statistical evaluation of spleen weight of 6 to 8 week-old male mice of different genotypes, (n ≥ 4). (B) Number of cells in spleen (n ≥ 6). (C) Ratio of spleen/body weight. Data were analyzed by one-way ANOVA with Tukey´s post-test. (* P < 0.05, ** P < 0.01 and *** P < 0.001). Each dot represents individual biological replicates. WT, Ormdl1-/- (O1KO), Ormdl3-/- (O3KO), Ormdl11-/-/Ormdl3-/- (O1/3dKO).

## Slide 4
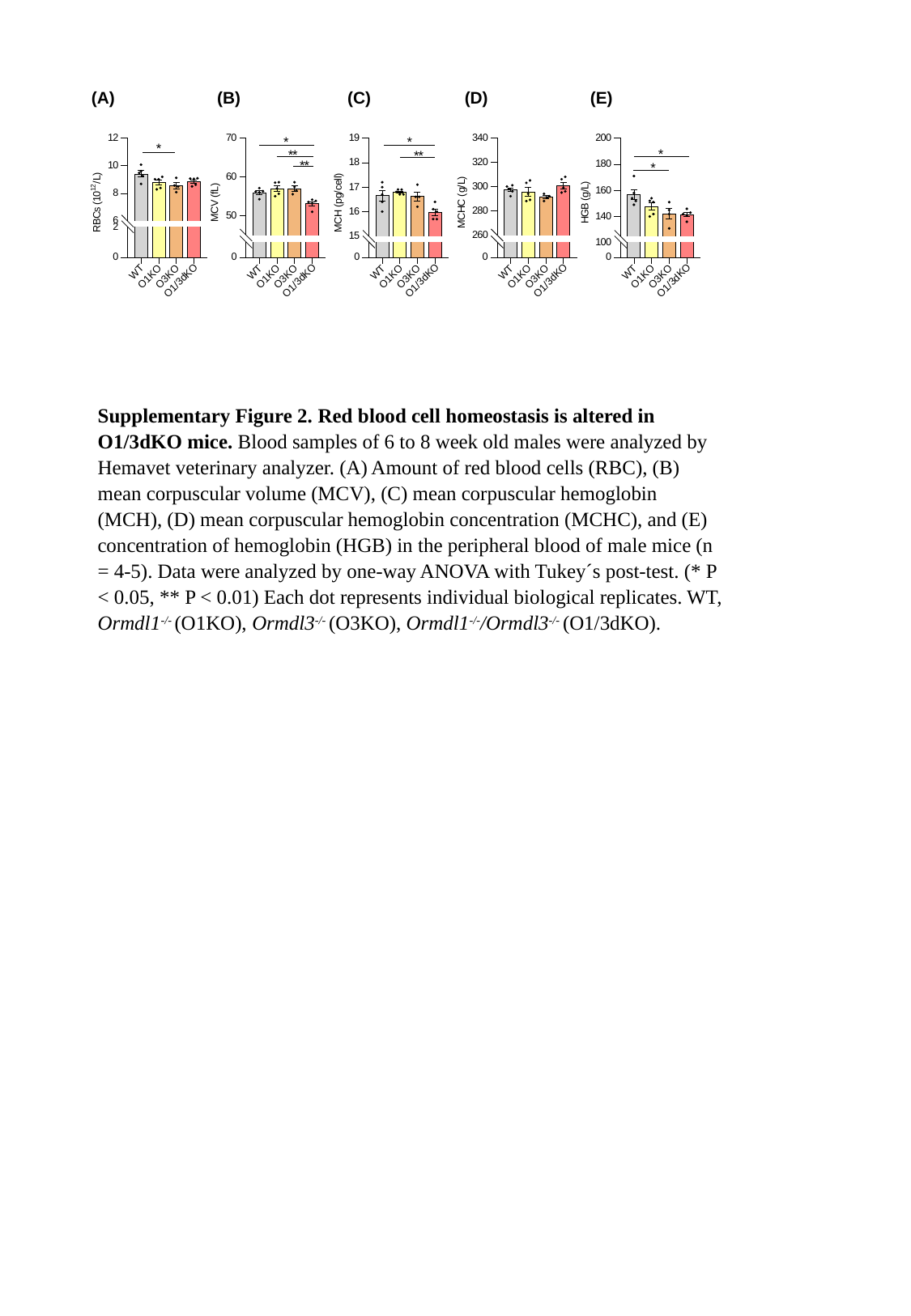

(A) (B) (C) (D) (E)
Supplementary Figure 2. Red blood cell homeostasis is altered in O1/3dKO mice. Blood samples of 6 to 8 week old males were analyzed by Hemavet veterinary analyzer. (A) Amount of red blood cells (RBC), (B) mean corpuscular volume (MCV), (C) mean corpuscular hemoglobin (MCH), (D) mean corpuscular hemoglobin concentration (MCHC), and (E) concentration of hemoglobin (HGB) in the peripheral blood of male mice (n = 4-5). Data were analyzed by one-way ANOVA with Tukey´s post-test. (* P < 0.05, ** P < 0.01) Each dot represents individual biological replicates. WT, Ormdl1-/- (O1KO), Ormdl3-/- (O3KO), Ormdl1-/-/Ormdl3-/- (O1/3dKO).

## Slide 5
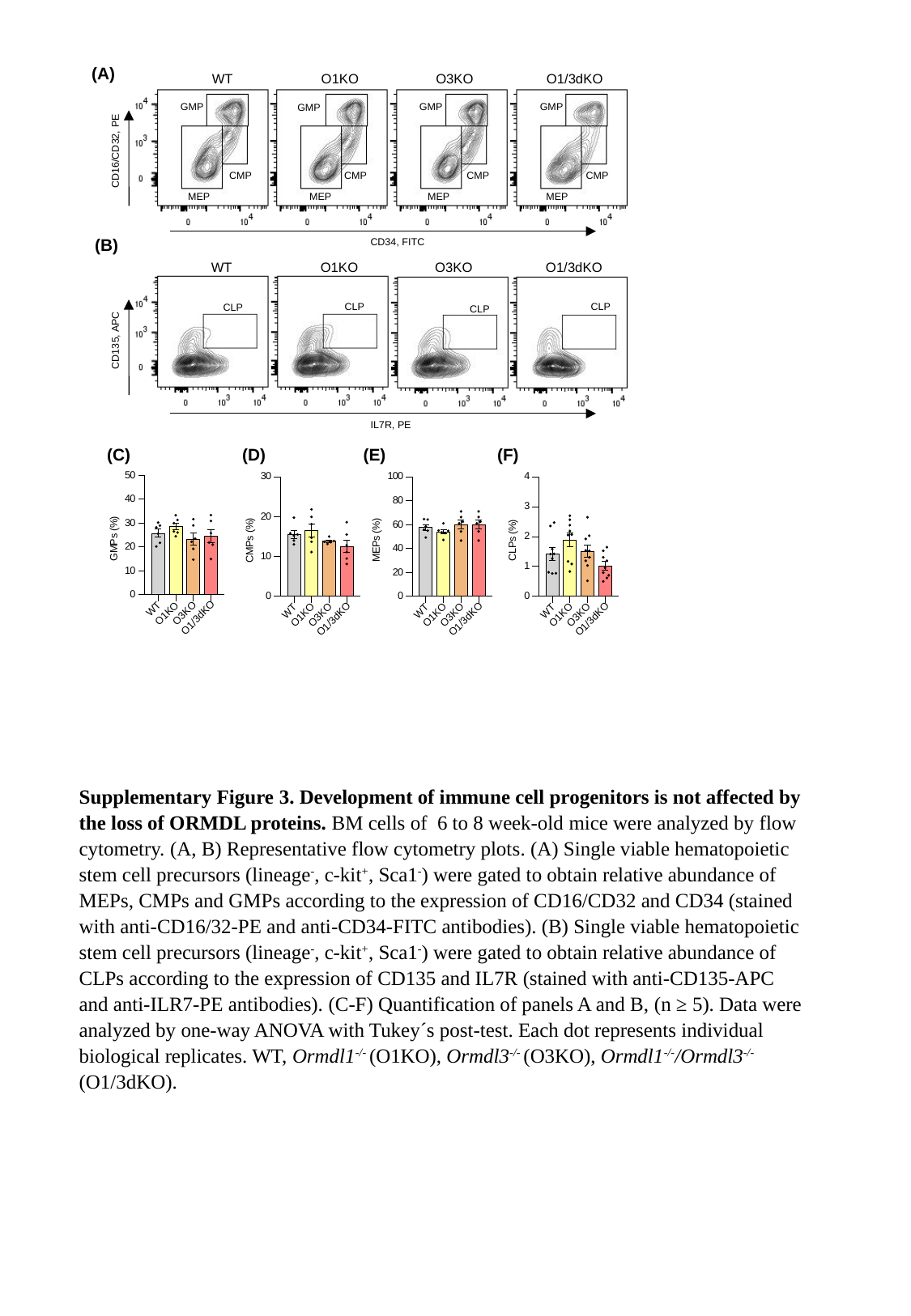

(A)
WT O1KO O3KO O1/3dKO
GMP
GMP
GMP
GMP
CMP
CMP
CMP
CMP
MEP
MEP
MEP
MEP
CD16/CD32, PE
(B)
CD34, FITC
WT O1KO O3KO O1/3dKO
CLP
CLP
CLP
CLP
CD135, APC
IL7R, PE
 (C) (D) (E) (F)
Supplementary Figure 3. Development of immune cell progenitors is not affected by the loss of ORMDL proteins. BM cells of 6 to 8 week-old mice were analyzed by flow cytometry. (A, B) Representative flow cytometry plots. (A) Single viable hematopoietic stem cell precursors (lineage-, c-kit+, Sca1-) were gated to obtain relative abundance of MEPs, CMPs and GMPs according to the expression of CD16/CD32 and CD34 (stained with anti-CD16/32-PE and anti-CD34-FITC antibodies). (B) Single viable hematopoietic stem cell precursors (lineage-, c-kit+, Sca1-) were gated to obtain relative abundance of CLPs according to the expression of CD135 and IL7R (stained with anti-CD135-APC and anti-ILR7-PE antibodies). (C-F) Quantification of panels A and B, (n ≥ 5). Data were analyzed by one-way ANOVA with Tukey´s post-test. Each dot represents individual biological replicates. WT, Ormdl1-/- (O1KO), Ormdl3-/- (O3KO), Ormdl1-/-/Ormdl3-/- (O1/3dKO).

## Slide 6
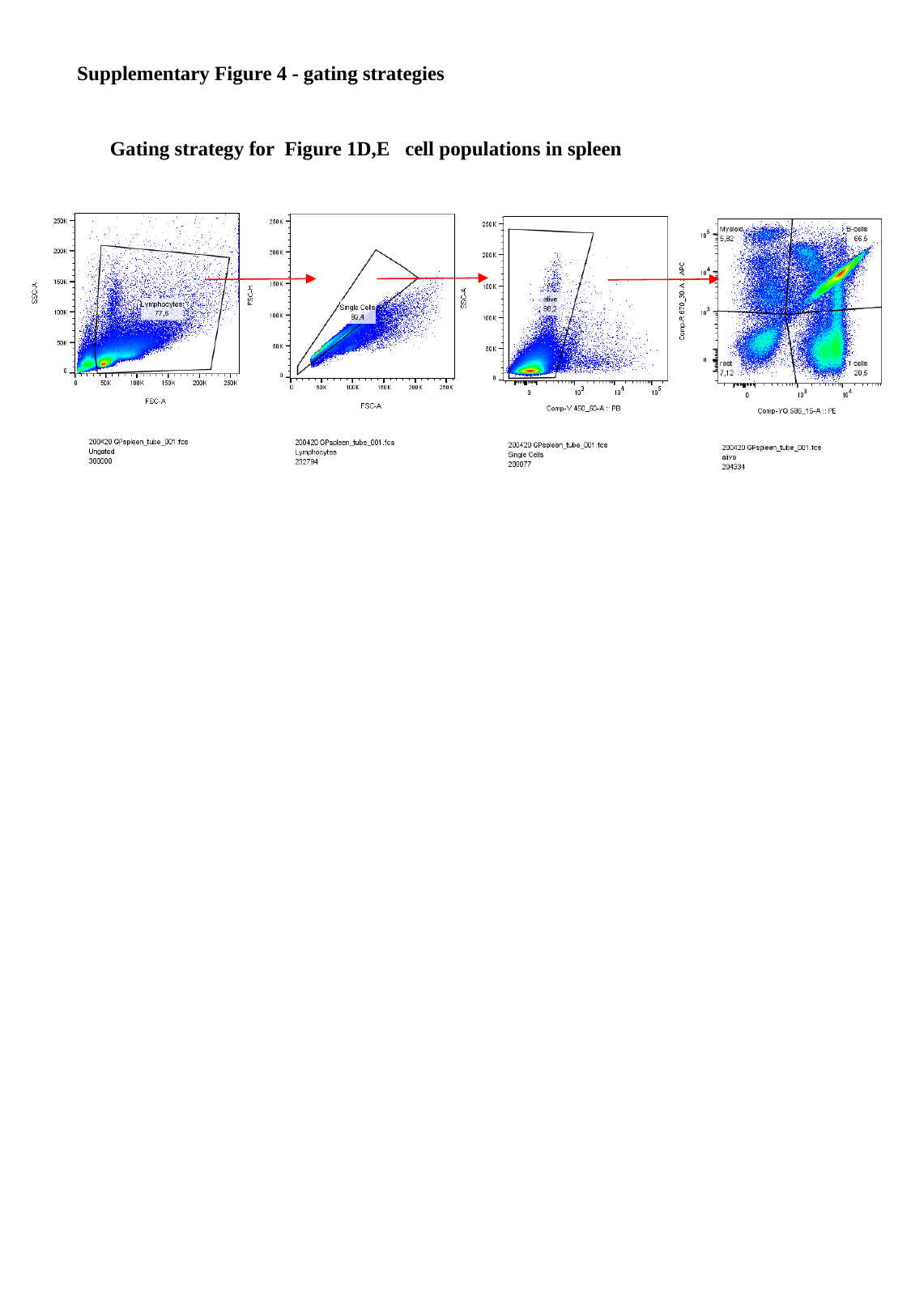

Supplementary Figure 4 - gating strategies
Gating strategy for Figure 1D,E cell populations in spleen

## Slide 7
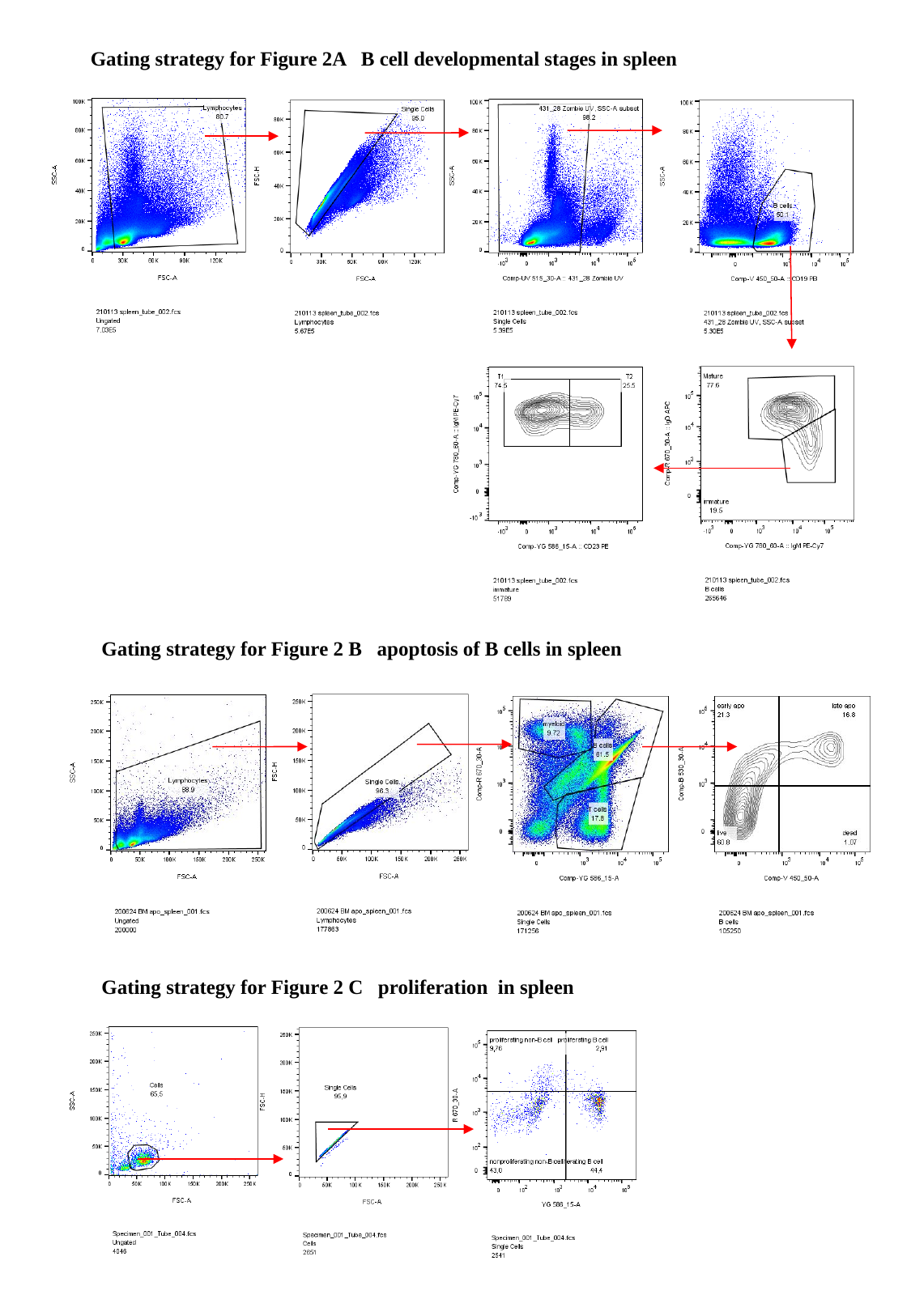

Gating strategy for Figure 2A B cell developmental stages in spleen
Gating strategy for Figure 2 B apoptosis of B cells in spleen
Gating strategy for Figure 2 C proliferation in spleen

## Slide 8
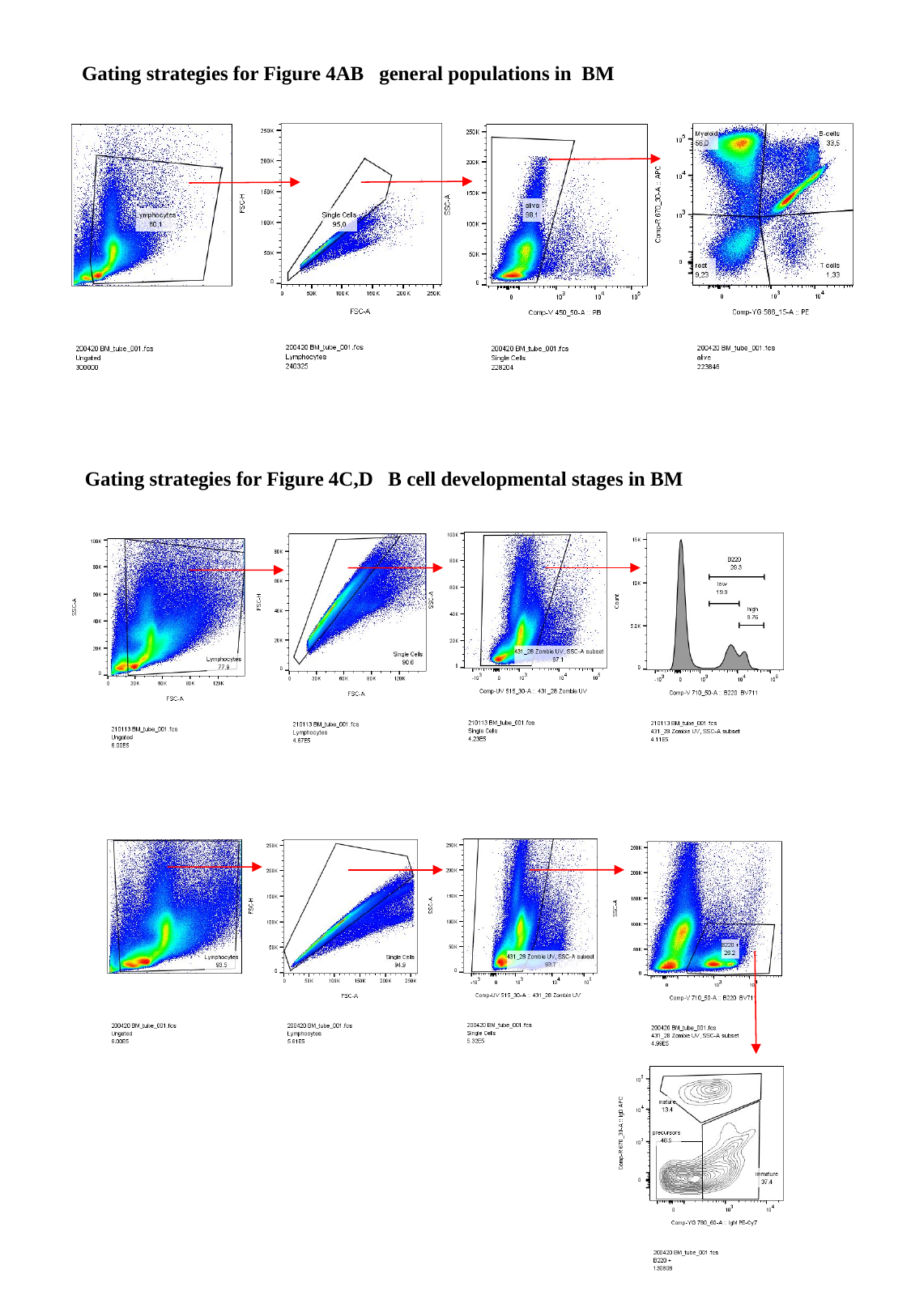

Gating strategies for Figure 4AB general populations in BM
Gating strategies for Figure 4C,D B cell developmental stages in BM

## Slide 9
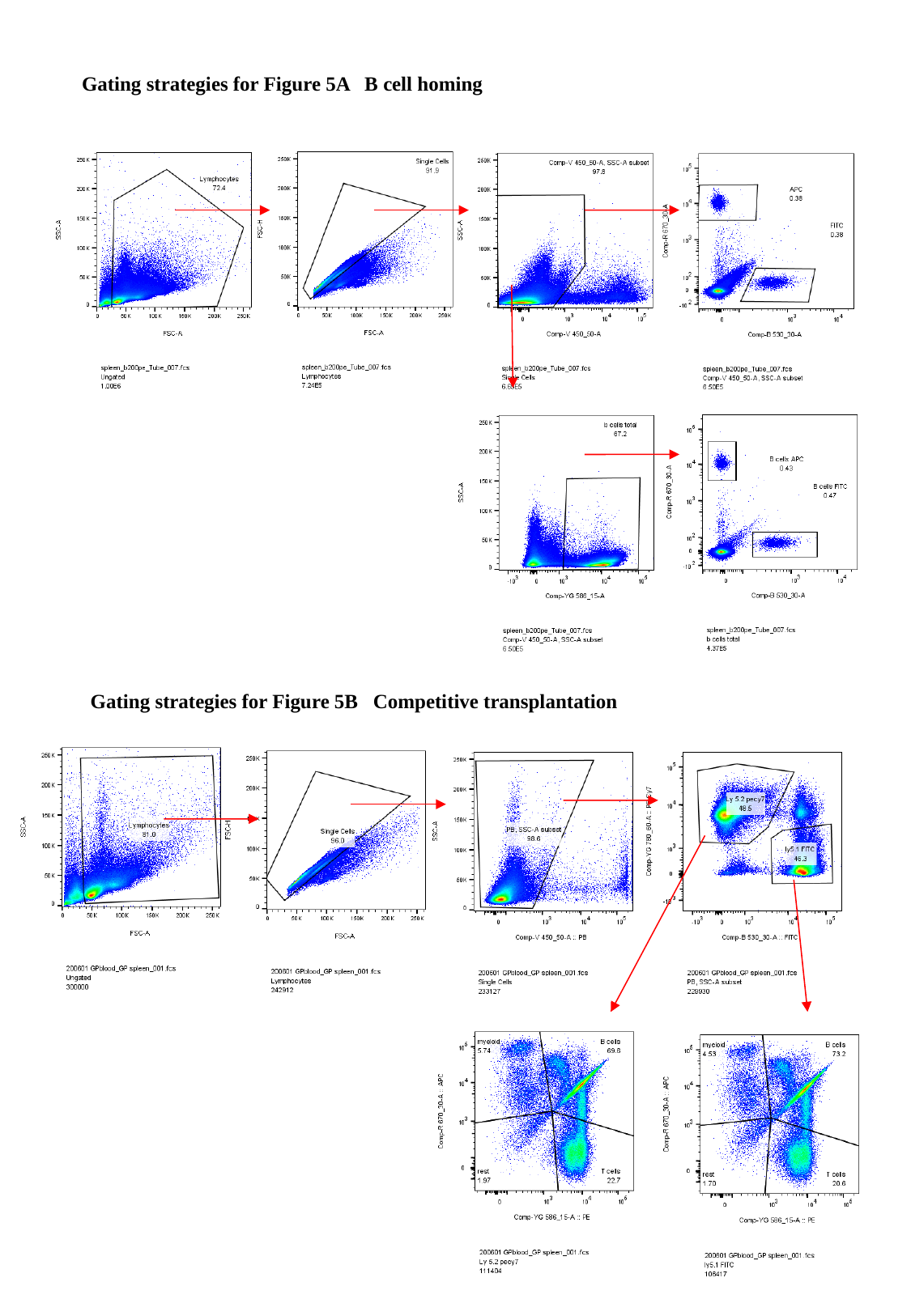

Gating strategies for Figure 5A B cell homing
Gating strategies for Figure 5B Competitive transplantation

## Slide 10
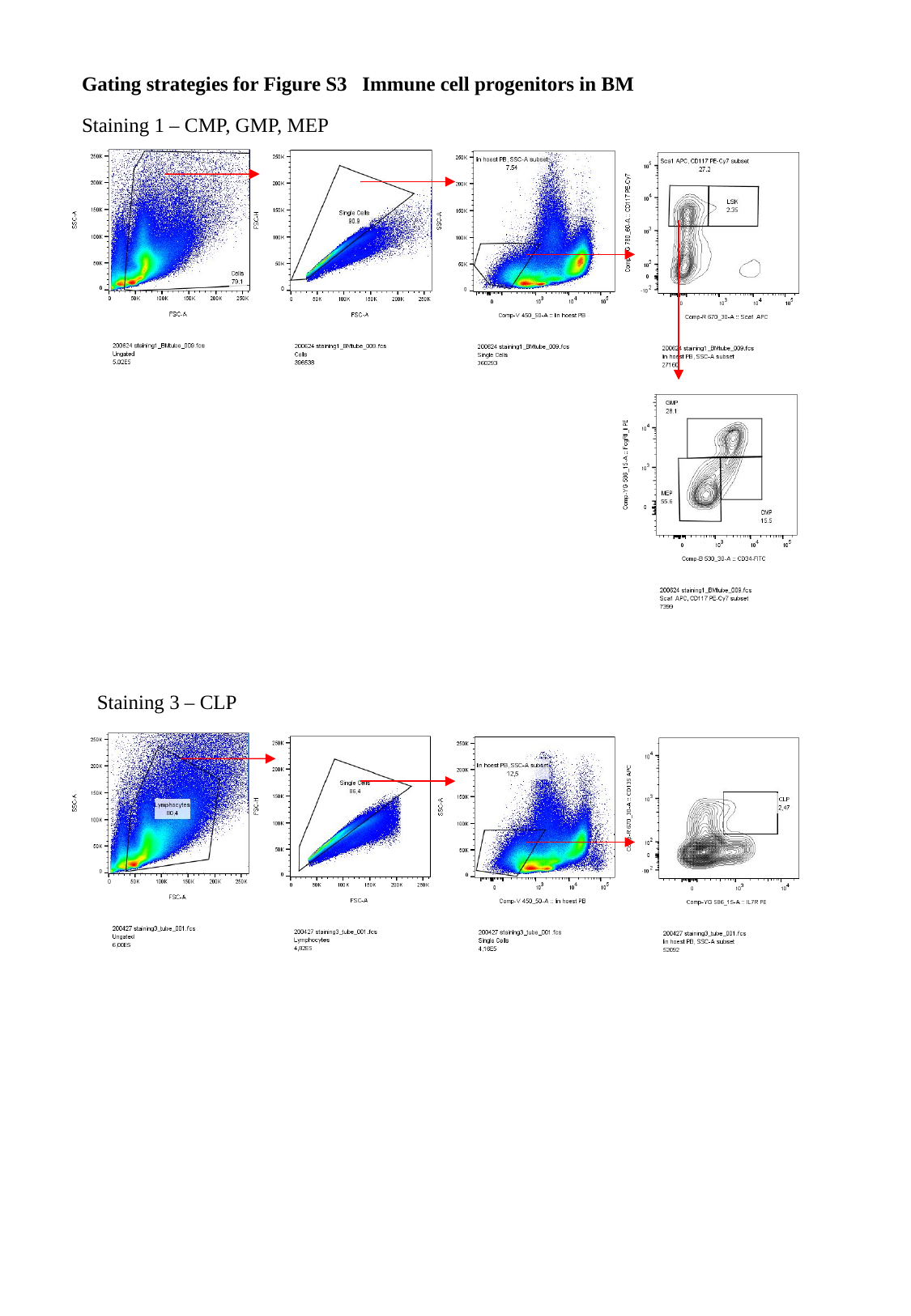

Gating strategies for Figure S3 Immune cell progenitors in BM
Staining 1 – CMP, GMP, MEP
Staining 3 – CLP
